# Capturing COPD heterogeneity: anomaly detection and parametric response mapping comparison for phenotyping on chest computed tomography

**DOI:** 10.3389/fmed.2024.1360706

**Published:** 2024-03-01

**Authors:** Silvia D. Almeida, Tobias Norajitra, Carsten T. Lüth, Tassilo Wald, Vivienn Weru, Marco Nolden, Paul F. Jäger, Oyunbileg von Stackelberg, Claus Peter Heußel, Oliver Weinheimer, Jürgen Biederer, Hans-Ulrich Kauczor, Klaus Maier-Hein

**Affiliations:** ^1^Division of Medical Image Computing, German Cancer Research Center (DKFZ), Heidelberg, Germany; ^2^Translational Lung Research Center Heidelberg (TLRC), German Center for Lung Research (DZL), Heidelberg, Germany; ^3^Medical Faculty, Heidelberg University, Heidelberg, Germany; ^4^National Center for Tumor Diseases (NCT), NCT Heidelberg, A Partnership Between DKFZ and Heidelberg University Medical Center, Heidelberg, Germany; ^5^Interactive Machine Learning Group (IML), German Cancer Research Center (DKFZ), Heidelberg, Germany; ^6^Helmholtz Imaging, German Cancer Research Center (DKFZ), Heidelberg, Germany; ^7^Division of Biostatistics, German Cancer Research Center (DKFZ), Heidelberg, Germany; ^8^Pattern Analysis and Learning Group, Radiation Oncology, Heidelberg University Hospital, Heidelberg, Germany; ^9^Diagnostic and Interventional Radiology, Heidelberg University Hospital, Heidelberg, Germany; ^10^Diagnostic and Interventional Radiology with Nuclear Medicine, Thoraxklinik at University Hospital, Heidelberg, Germany; ^11^Faculty of Medicine, University of Latvia, Riga, Latvia; ^12^Faculty of Medicine, Christian-Albrechts-Universität zu Kiel, Kiel, Germany

**Keywords:** chronic obstructive pulmonary disease, computed tomography, GOLD, airway disease, emphysema, artificial intelligence, anomaly detection

## Abstract

**Background:**

Chronic obstructive pulmonary disease (COPD) poses a substantial global health burden, demanding advanced diagnostic tools for early detection and accurate phenotyping. In this line, this study seeks to enhance COPD characterization on chest computed tomography (CT) by comparing the spatial and quantitative relationships between traditional parametric response mapping (PRM) and a novel self-supervised anomaly detection approach, and to unveil potential additional insights into the dynamic transitional stages of COPD.

**Methods:**

Non-contrast inspiratory and expiratory CT of 1,310 never-smoker and GOLD 0 individuals and COPD patients (GOLD 1–4) from the COPDGene dataset were retrospectively evaluated. A novel self-supervised anomaly detection approach was applied to quantify lung abnormalities associated with COPD, as regional deviations. These regional anomaly scores were qualitatively and quantitatively compared, per GOLD class, to PRM volumes (emphysema: PRM^Emph^, functional small-airway disease: PRM^fSAD^) and to a Principal Component Analysis (PCA) and Clustering, applied on the self-supervised latent space. Its relationships to pulmonary function tests (PFTs) were also evaluated.

**Results:**

Initial t-Distributed Stochastic Neighbor Embedding (t-SNE) visualization of the self-supervised latent space highlighted distinct spatial patterns, revealing clear separations between regions with and without emphysema and air trapping. Four stable clusters were identified among this latent space by the PCA and Cluster Analysis. As the GOLD stage increased, PRM^Emph^, PRM^fSAD^, anomaly score, and Cluster 3 volumes exhibited escalating trends, contrasting with a decline in Cluster 2. The patient-wise anomaly scores significantly differed across GOLD stages (*p* < 0.01), except for never-smokers and GOLD 0 patients. In contrast, PRM^Emph^, PRM^fSAD^, and cluster classes showed fewer significant differences. Pearson correlation coefficients revealed moderate anomaly score correlations to PFTs (0.41–0.68), except for the functional residual capacity and smoking duration. The anomaly score was correlated with PRM^Emph^ (*r* = 0.66, *p* < 0.01) and PRM^fSAD^ (*r* = 0.61, *p* < 0.01). Anomaly scores significantly improved fitting of PRM-adjusted multivariate models for predicting clinical parameters (*p* < 0.001). Bland–Altman plots revealed that volume agreement between PRM-derived volumes and clusters was not constant across the range of measurements.

**Conclusion:**

Our study highlights the synergistic utility of the anomaly detection approach and traditional PRM in capturing the nuanced heterogeneity of COPD. The observed disparities in spatial patterns, cluster dynamics, and correlations with PFTs underscore the distinct – yet complementary – strengths of these methods. Integrating anomaly detection and PRM offers a promising avenue for understanding of COPD pathophysiology, potentially informing more tailored diagnostic and intervention approaches to improve patient outcomes.

## 1 Introduction

Chronic obstructive pulmonary disease (COPD) remains a major global health burden, ranking as the third leading cause of mortality worldwide (1). Characterized by progressive airflow limitation, COPD predominantly arises from prolonged exposure to harmful airborne particles, particularly in individuals with a history of smoking (2, 3). Early detection and accurate phenotyping of COPD are paramount, as timely intervention, including smoking cessation and appropriate treatments, may slow disease progression and improve patient outcomes. Although pulmonary function testing (PFT) and, in particular, gold standard spirometry, play a central role in COPD diagnosis, its ability to detect early-stage disease and reliably characterize its heterogeneity remains limited (4, 5).

Recent advances in imaging technology, particularly computed tomography (CT), have been instrumental to gain insights into COPD pathophysiology, by quantifying emphysema and small-airway disease. Several emphysema quantification methods are based on measuring the relative area of the lungs below a specific Hounsfield unit (HU) threshold, i.e., low attenuation areas (LAA), on inspiratory CT scans, showing significant correlations with pulmonary function test (PFT) parameters (6–8).

While large airways down to the first sub-segmental generations are clearly visible on CT, smaller, more distal subsegmental airways and respiratory bronchioles cannot be detected unless becoming more conspicuous due to mucus retention or peribronchial inflammation. However, small airways disease can be indirectly assessed and quantified with CT scans in expiration, when small airway obstruction results in air trapping (8). Several methods, including measuring the LAA below −856 HU (LAA-856) on expiratory scans, have been proposed for evaluating air trapping (8). However, the optimal CT-based method to assess small-airway disease remains a subject of ongoing debate. In recent years, parametric response mapping (PRM) has emerged as a novel approach to phenotyping COPD by utilizing both inspiration and expiration CT scans (9). PRM allows differentiation between emphysematous and non-emphysematous air trapping regions. For small-airway disease, PRM identifies lung areas with densities greater than or equal to −950 HU on inspiration CT and less than −856 HU on expiration CT. However, PRM’s current method for small-airway disease assessment focuses on slight dynamic density changes within each voxel and may not fully consider emphysema’s potential contribution to air trapping assessment. As it is a mutually exclusive voxel-wise method, each voxel is assigned exclusively to either the emphysematous or non-emphysematous category, potentially oversimplifying the intricate interplay between emphysema and small-airway disease. Additionally, its method’s dependence on fixed thresholds introduces limitations in capturing variations across diverse patient populations, while the reliance on registration may be susceptible to specific methods and anatomical variations. Moreover, the sensitivity to CT-protocol variations poses a challenge in ensuring consistent and reproducible results across different imaging settings.

With advances in deep learning (DL) based artificial intelligence (AI), several algorithms have been developed to target the direct interpretation of CT scans, without the need to pre-define COPD features of interest. While the majority relies on supervised learning (10–14), self-supervised (15) and unsupervised methods (16) have been gaining a lot of attention. Their ability to capture complex disease heterogeneity without the need for explicit annotation, makes them particularly advantageous in scenarios where manual labeling may be challenging or subjective. Moreover, these methods have the potential to discover novel disease subtypes or manifestations that may not be predefined in the training dataset, enhancing their adaptability to the diverse and evolving nature of COPD.

In particular, Almeida et al. (15) introduced a self-supervised DL anomaly-detection approach that identifies COPD lung regions as anomalies, reflecting the varied manifestations of COPD across different phenotypes, including large and small airway disease, parenchymal scars, and emphysema. The method harnesses informative latent representations and a generative model to identify deviations from the distribution of normal-appearing lung regions of individuals without airflow obstruction. These deviations are then presented as a lung anomaly map with anomaly scores. Importantly, it has demonstrated its value in distinguishing normal individuals from those with COPD and predicting lung function decline (17).

While being a promising tool for the assessment of the severity of COPD, the regional anomaly scores derived from this method have not been further explored. Hence, our study aims to compare the quantitative and spatial relationships between PRM functional small airway disease and emphysema measurements to regional anomaly scores. This can further provide critical insights into COPD heterogeneity and implications for personalized patient care.

## 2 Materials and methods

**Figure 1:**
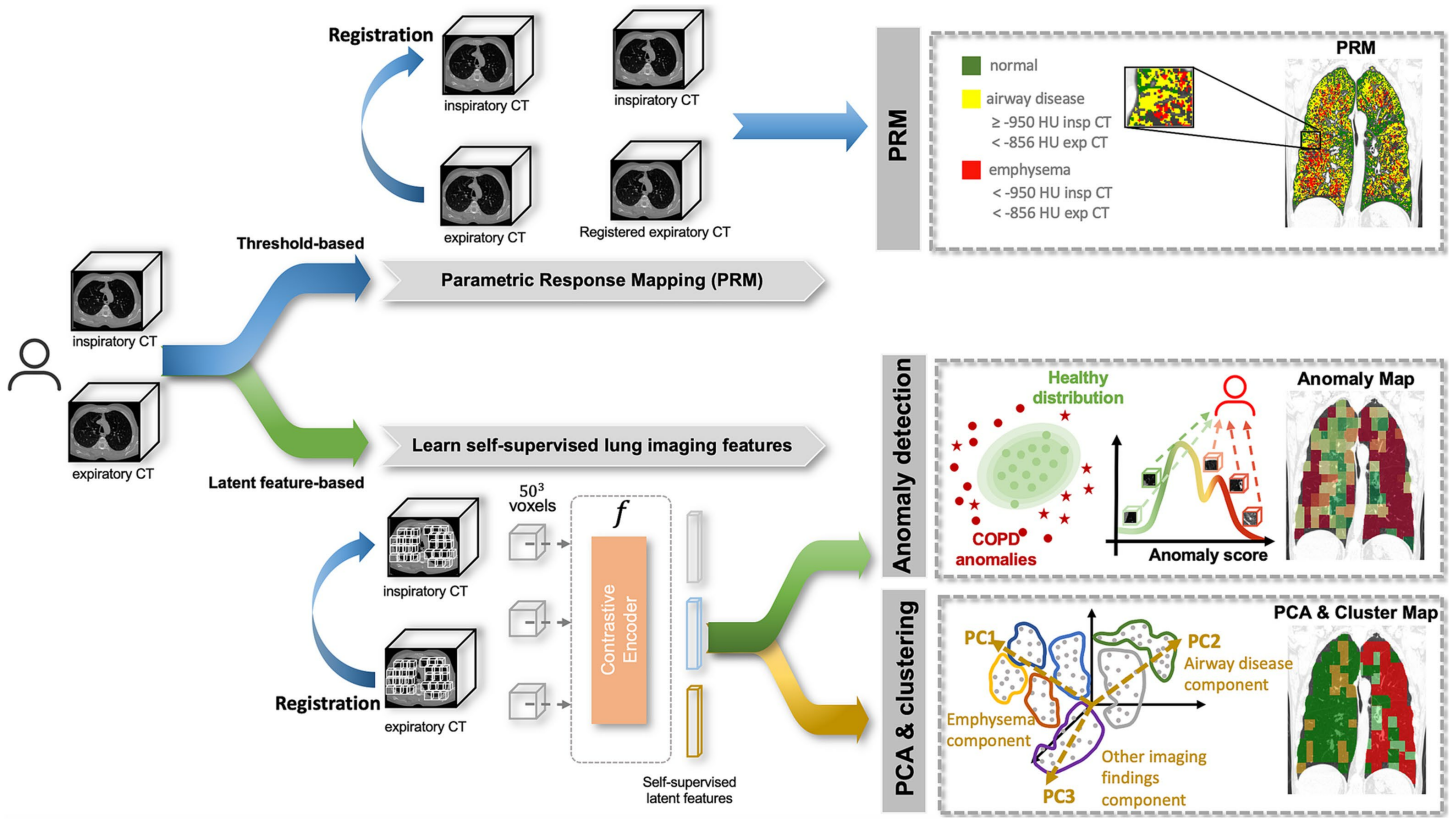
Methodology pipeline. A subject is composed of paired inspiratory and expiratory CT scans which are analyzed by 3 methods: (1) Parametric response mapping (PRM), which relies on the spatial alignment of the two scans and defines emphysema and airway disease areas based on strict thresholds; (2) Anomaly detection, which attributes region-wise anomaly scores based on the distribution of normal lung features, defined by a self-supervised contrastive method; (3) Principal Component Analysis (PCA) & Clustering, which applies dimensionality reduction (PCA) to the latent features from the self-supervised contrastive method to find stable clusters. All three methods produce lung maps, which were then compared qualitatively and quantitively.

The objective of our study, depicted in Figure 1, was to perform a quantitative and qualitative comparison of spatial relationships between traditional (1) Parametric response mapping (PRM) volumes and the (2) Self-Supervised Anomaly Detection method (15, 17). Our hypothesis posits that the anomaly scores derived from self-supervised learning align with PRM volumes, and their associations with clinical metrics are comparable. To enhance our understanding of what the self-supervised method learns, we also employ (3) Principal Component Analysis (PCA) & Clustering on the self-supervised representation level. This approach identifies stable clusters, allowing us to compare them with the aforementioned two methods.

### 2.1 Study cohort

In the present study, we utilized the Genetic Epidemiology of COPD (COPDGene) study. The COPDGene study (ClinicalTrials.gov Identifier NCT00608764) recruited never-smoker controls and current and former smokers, who had a smoking history of ≥10 pack-years. The enrollment period was conducted between 2008 and 2011, targeting individuals aged 45–80 years.

Comprehensive assessments were performed, including paired chest CT scans during inspiration (Insp) and expiration (Exp), pulmonary function tests (PFT), and questionnaire evaluations. Specific details regarding the CT acquisition protocol can be found in Almeida et al. (17).

Ethical approval was obtained, and written consent was acquired from all participants after the study protocol received approval from the respective clinical center’s review board. The inclusion and exclusion criteria were previously described in Almeida et al. (15, 17).

In accordance with the established protocol defined in Almeida et al. (15, 17), the dataset was partitioned into distinct sets for training, evaluation and testing.

### 2.2 Parametric response mapping

Parametric response mapping (PRM) was applied to the paired Insp and Exp CT scans as described in the original work (9) of the test set. This process categorized the lung parenchyma into functional small-airway disease (PRM^fSAD^), emphysema (PRM^Emph^), and normal lung (PRM^Normal^). To minimize the contribution of airways and vessels, specific minimum and maximum attenuation values were defined for both scans. PRM^Emph^ was defined by voxels between −1,000 HU and − 950 HU in the inspiratory CT and between −1,000 HU and −856 HU in the expiratory CT scan. PRM^fSAD^ was defined by voxels between −950 HU and −810 HU in the inspiratory CT and − 1,000 HU and −856 HU in the expiratory CT. Lastly, PRM^Normal^ was defined by voxels between −950 HU and −810 HU in the inspiratory CT and −856 and −500 HU in the expiratory CT scan. The top section of Figure 1 illustrates this step.

Following the PRM analysis, the subsequent sections detail the application of (2) Anomaly Detection and (3) PCA & Clustering, both involving two common steps: quantitative pre-processing and extraction of self-supervised latent features.

### 2.3 Pre-processing

#### 2.3.1 Quantitative pre-processing

After segmentation of the lung parenchyma, 3D Regions of Interest (ROIs) of size 50 × 50 × 50 voxels (covering >70% of the lung parenchyma) were extracted from paired Insp and registered Exp (ExpR) CT scans, with 20% patch-overlapping, as previously described in Almeida et al. (15, 17).

#### 2.3.2 Extraction of self-supervised latent features

Following the extraction of 3D patches, the objective was to create a representation per patch that encapsulated relevant information related to its imaging features. This representation vector, of size 1 × 512, was later employed for both (1) Anomaly Detection and (2) PCA & Clustering. The self-supervised learning strategy described in Almeida et al. (15, 17) was employed, where a subset of the COPDGene subjects was used to train a self-supervised contrastive model, without using any labels. This model is based on the idea of learning representations that maximize the agreement between differently augmented views of the same region via a contrastive loss, i.e., attracting regions that look similar and repel the ones that do not. Based on this, informative latent representations were generated per patch, as illustrated in Figure 1.

### 2.4 Visualization of the latent space: t-distributed stochastic neighbor embedding

For understanding the information conveyed in the self-supervised latent features of each region and for visualization purposes, t-Distributed Stochastic Neighbor Embedding (t-SNE) (18) was applied as a non-linear dimensionality reduction technique. t-SNE is a common dimensionality reduction technique that maps high-dimensional data into a lower-dimensional space while preserving the pairwise similarities between data points. In order to preserve better global structure, non-standard affinity methods were initialized with the evaluation set, and later transformed to the test set. This was implemented using the openTSNE package (19).

### 2.5 Anomaly detection

Subsequently, having the patch-level latent representations, the anomaly detection model was applied, as illustrated in Figure 1.

The model aimed to quantify the degree to which a specific region or patient deviates from the pre-defined “normality,” as defined in Almeida et al. (17). This normative baseline was defined by lung regions with less than 1% emphysema from individuals without airflow obstruction (never-smoker controls and GOLD0). The distribution of these “normal/healthy” latent features, as derived from the self-supervised contrastive approach, serves as the reference for the model. Region, i.e., patch-wise, anomaly scores are computed using the negative log-likelihood. Patient-level anomaly scores are subsequently obtained by aggregating scores from all regions. Further details can be found in Almeida et al. (15, 17).

For visualization purposes of the anomaly lung map, min-max normalization was applied to the anomaly scores, corresponding to the 5th and 95th percentiles of the dataset.

### 2.6 Principal component analysis and clustering

Principal Component Analysis (PCA) & Clustering served as a direct comparative approach to the anomaly detection model, as both operate on the self-supervised latent features of the test set. These representations were obtained by a region-similarity approach, which grouped similar patterns together (whether region- or intensity-wise). The objective with the PCA & Clustering was to explore and identify clusters of regions sharing similar characteristics within the informative latent features, and directly compare it to the anomaly detection method and to the reference PRM volumes.

#### 2.6.1 PCA on the self-supervised latent features

PCA was applied to the latent vectors of each region of the test set, in order to mitigate multicollinearity along the 512 features. The eigenvectors of the covariance matrix were analyzed. Then to reduce the dimension space, the dataset was projected onto the first few uncorrelated principal components, representing dominant eigenvectors of the covariance matrix. The optimal number of Principal Components (PCs) was decided based on Horn’s parallel analysis (20), through a Scree Plot, and based on the Kaiser Criterion.

#### 2.6.2 Clustering the PCA

Once determined the subset of PCs to retain, cluster analysis was conducted on the dataset. Various clustering methods, including K-means and Gaussian finite mixture model-based methods were compared using Silhouette, Davies-Bouldin and Calinski and Harabasz scores (21). The Silhouette score aimed for maximization, providing a measure of the separation among different clusters. Davies-Bouldin, targeted for minimization, assessed the similarity of each cluster to its next closest neighbor. Calinski and Harabasz, also targeted for minimization, estimated the cohesion and separation of points within a cluster, based on the distance between cluster centroids.

Based on these scores, the optimal clustering method and number of clusters were determined and applied for further analysis. Further information is provided in the Supplementary materials. However, it’s important to note that these clusters were primarily generated for comparison purposes. They serve as a reference point to assess the efficacy of the anomaly detection method, since both employ the same self-supervised latent features.

### 2.7 Statistical analysis

Comparisons among PRM volumes, Patient-wise Anomaly Scores and each class of Anomaly Region Clusters were made according to the Global Initiative for Chronic Obstructive Lung Disease (GOLD) stages using the Jonckheere-terpstra test. A post-hoc Tukey-test was then applied for multiple pairwise-comparisons between GOLD stages.

The relationships between pulmonary function tests, clinical data and each PRM class, anomaly scores and cluster groups were evaluated through Pearson’s correlation coefficient. Confidence intervals (CI) were calculated using bootstrap resampling method on 10,000 samples. Pulmonary function tests and clinical data included parameters such as FEV1% predicted, FEV1/FVC, Functional Residual Capacity (FRC), Total Lung Capacity (TLC), FRC/TLC, BODE index (body mass index, air-flow obstruction, dyspnea, exercise capacity), St. George’s Respiratory Questionnaire (SGRQ), the 6-min-walking-test (6MWT) and smoking duration. Correlations were interpreted as follows: 0.00–0.10 (negligible), 0.10–0.39 (weak), 0.40–0.69 (moderate), 0.70–0.89 (strong), and 0.90–1.00 (very strong) (22). Differences between correlation coefficients were assessed via the R package “cocor” (23), utilizing the Zou et al. (24) method. This involved calculating the difference between correlation coefficients for each pair of groups and determining a 95% confidence interval (CI) for that difference. If the CI included zero, the null hypothesis that the two correlations are equal was retained; if the CI did not include zero, the null hypothesis was rejected.

Linear mixed effects models (LMM) were utilized to predict clinical parameters based on the PRM volumes, with adjustments made for relevant covariates including age, gender, body mass index (BMI), smoking status (0: never-smoker control, 1: former smoker, and 2: current smoker), smoking duration, and a random term for the study site. To assess the contribution of the anomaly score beyond morphological lung changes in predicting clinical variables (FEV1%, FEV/FVC, FRC, TLC, FRC/TLC, BODE, SGRQ, and 6MWT), it was then introduced as an additional predictor in the PRM-adjusted LMM models. The overall conditional coefficient of determination (R2), adjusted for the number of regressors, was reported. Models were compared through the likelihood ratio test of nested models.

The agreements between PRM relative volumes and volumes obtained from the Cluster groups were assessed using the Bland–Altman method (25). A *p*-value of <0.05 was considered as statistically significant and adjustments were made for multiple comparisons, using the Holm-Bonferroni method, when applicable.

All statistical analyses were performed using R software version 4.3.4 (R Foundation for Statistical Computing, Vienna, Austria).

## 3 Results

Patient characteristics are summarized in Table 1.

**Table 1:** Subject demographics (sex, age, body mass index [BMI]), functional parameters (post-bronchodilator forced expiratory volume in one second [FEV1%_pred]; FEV1/Forced Vital Capacity [FVC]; Functional Residual Capacity [FRC]; Total Lung Capacity [TLC]; FRC/TLC; Body-Mass Index, Airflow Obstruction, Dyspnea, and Exercise Capacity Index [BODE]; St. George‘s Respiratory Questionnaire [SGRQ]; 6-min walking test [6MWT]), smoking duration and low-attenuation (LAA) percentages [LAA-950% and LAA-856%], for the evaluation population, from the COPDGene dataset.

	Never-smoker controls (*N* = 29)	GOLD 0 (*N* = 538)	GOLD 1 (*N* = 128)	GOLD 2 (*N* = 315)	GOLD 3 (*N* = 195)	GOLD 4 (*N* = 105)	*p*-value
Sex [f/m]	11/18	280/258	74/54	161/154	116/79	50/55	0.011
Age (y)	62.2 ± 9.1	59.5 ± 8.3	63.2 ± 8.7*	64.8 ± 8.6	66.2 ± 7.9	65.4 ± 7.0	<0.001
BMI	27.8 ± 4.3	28.6 ± 5.6	27.1 ± 4.2	28.5 ± 6.0	28.4 ± 6.5	25.4 ± 4.8*	<0.001
FEV1%_pred	100.6 ± 13.2	96.4 ± 10.7	90.4 ± 9.0*	65.8 ± 8.8*	39.4 ± 5.5*	23.2 ± 4.2*	<0.001
FEV1/FVC	0.8 ± 0.1	0.8 ± 0.0	0.6 ± 0.0*	0.6 ± 0.1*	0.4 ± 0.1*	0.3 ± 0.1*	<0.001
FRC	2.5 ± 0.6	2.9 ± 0.7	3.4 ± 0.9*	3.5 ± 0.9	4.5 ± 1.2*	5.1 ± 1.3*	<0.001
TLC	5.2 ± 1.4	5.7 ± 1.2	6.4 ± 1.5*	5.9 ± 1.3	6.3 ± 1.6	6.6 ± 1.4	<0.001
FRC/TLC	0.5 ± 0.1	0.5 ± 0.1	0.5 ± 0.1	0.6 ± 0.1*	0.7 ± 0.1*	0.8 ± 0.1*	<0.001
BODE	0.1 ± 0.3	0.3 ± 0.7	0.5 ± 0.9	1.4 ± 1.3*	3.9 ± 1.4*	5.5 ± 1.2*	<0.001
SGRQ	4.3 ± 8.1	13.0 ± 15.6	17.5 ± 17.9	31.0 ± 20.8*	45.5 ± 18.0*	55.8 ± 16.6*	<0.001
6MWT (meters)	539.4 ± 88.7	476.3 ± 100.4	468.9 ± 105.4	415.6 ± 109.0*	344.5 ± 118.8*	267.4 ± 102.5*	<0.001
Smoking duration (y)	-	32.8 ± 10.9	38.3 ± 10.2*	40.3 ± 9.5	41.3 ± 8.7	40.3 ± 8.5	<0.001
LAA-950%	1.9 ± 2.0	2.6 ± 2.9	5.1 ± 5.1	8.3 ± 8.4	15.5 ± 12.2	27.9 ± 13.4	<0.001
LAA-856%	9.4 ± 6.8	12.0 ± 9.8	19.9 ± 11.2	28.7 ± 15.9*	47.5 ± 17.3*	63.7 ± 11.8*	<0.001

Attenuation percentages were measured by VIDA Diagnostics. All data are given as mean ± standard deviation. For some cases, data was not available: 17 for 6MWT, 17 for BODE, 76 for FRC, 3 for TLC, 61 for FRC/TLC, 3 for LAA-950%, 76 for LAA-856%. *p-*value was obtained by the Jonckheere-Terpstra test or a logistic regression, when applicable. * denotes *p* < 0.05 vs. previous GOLD stage for the post-hoc Tukey-test comparison.

### 3.1 Visualization of the latent space: t-distributed stochastic neighbor embedding

**Figure 2:**
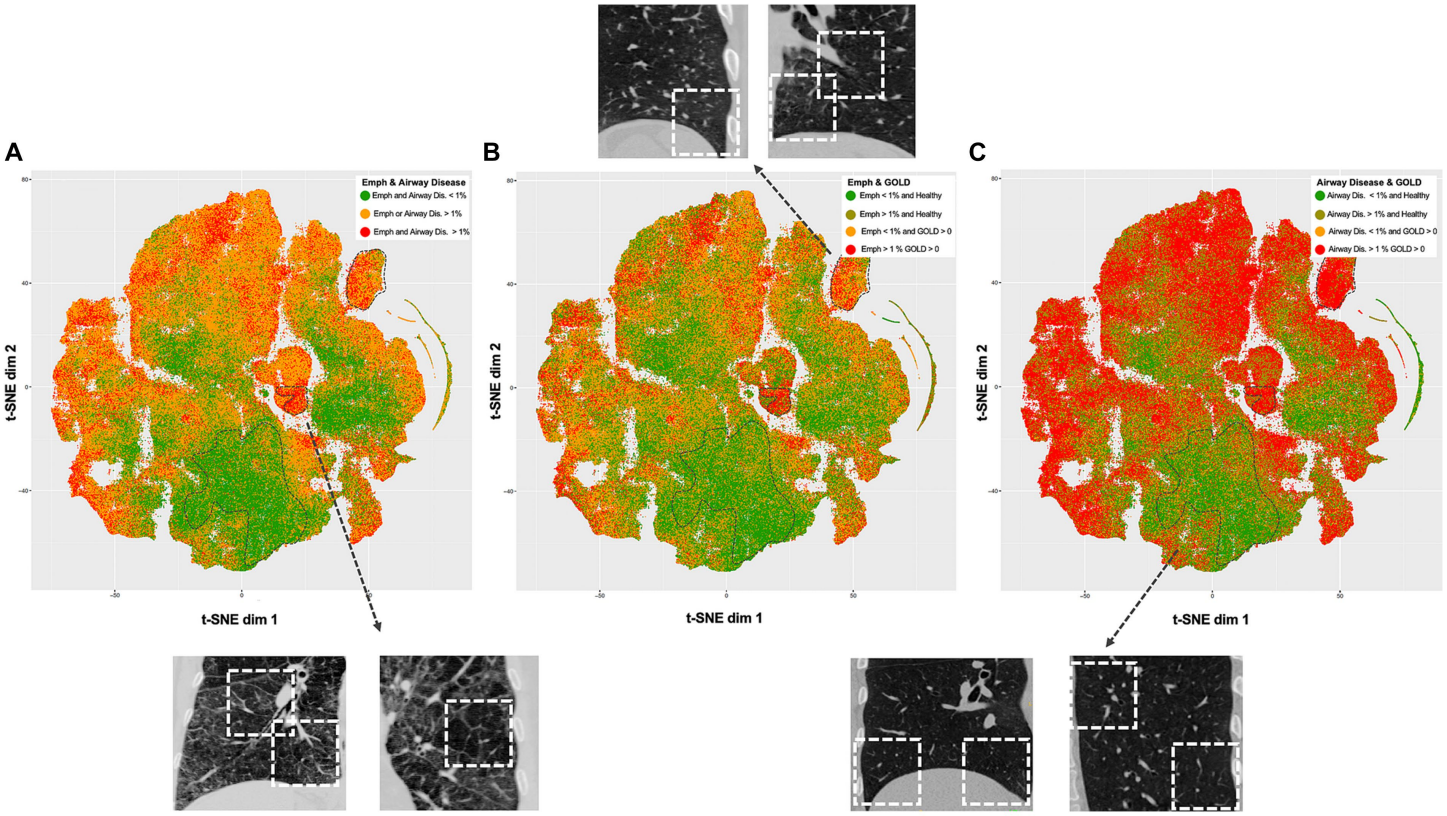
t-Distributed Stochastic Neighbor Embedding (t-SNE) visualizations of the self-supervised contrastive latent space vectors. Each dot represents a region (3D ROI) or more specifically, an embedding of its latent representation into a two-dimensional space, and its color represents a clinical or radiological characteristic (GOLD, emphysema and air trapping measures at the patch-level). The dotted regions in the t-SNE maps emphasize distinct groups (bottom – healthy, center right and upper right – diseased), serving as illustrative examples. For each dotted region, several examples are provided to illustrate representative inspiratory patches extracted from those dotted regions. Readers are guided to recognize consistent groups positioning across visualizations, enhancing overall interpretation of the t-SNE plots. **(A)** Visualization is colored by regions with and without emphysema and airway disease. **(B)** Visualization is colored by regions with less than 1% emphysema from healthy individuals (green), regions with more than 1% emphysema from healthy individuals (dark green), regions with less than 1% emphysema from COPD individuals (yellow) and regions with more than 1% emphysema from COPD individuals (red). **(C)** Same as in B but related to airway disease. Healthy individuals were defined as controls and GOLD 0, while COPD individuals as GOLD 1, 2, 3, or 4.

The comprehensive representation of the lung regions in the latent space, achieved through self-supervised contrastive learning, is visually depicted using t-SNE (Figure 2). Each point represents a feature vector (1 × 512) per lung region (3D patch). A clear separation between regions with and without emphysema and gas trapping is consistently illustrated. Also, regions exhibiting less than 1% Emphysema and less than 1% Air Trapping are distinctly discernable from diseased regions. Notably, factors such as gender information do not contribute to the separation of these latent features (Supplementary Figure S4).

### 3.2 PCA and cluster analysis

PCA applied to the region-wise latent vectors determined that 85 factors should be retained, meeting the criteria of obtained eigenvalues surpassing those from random data, as per Horn’s parallel analysis and the Kaiser Criterion (Supplementary Figure S1). Supplementary Figure S2 provides a comparison between the number of clusters and the method applied to the 85 retained PCs. Cluster analysis identified four stable clusters (Clusters 1, 2, 3, and 4) through K-means with a mini-batch size of 1,000. More details about the clusters are available in the Supplementary materials.

### 3.3 Qualitative results

**Figure 3:**
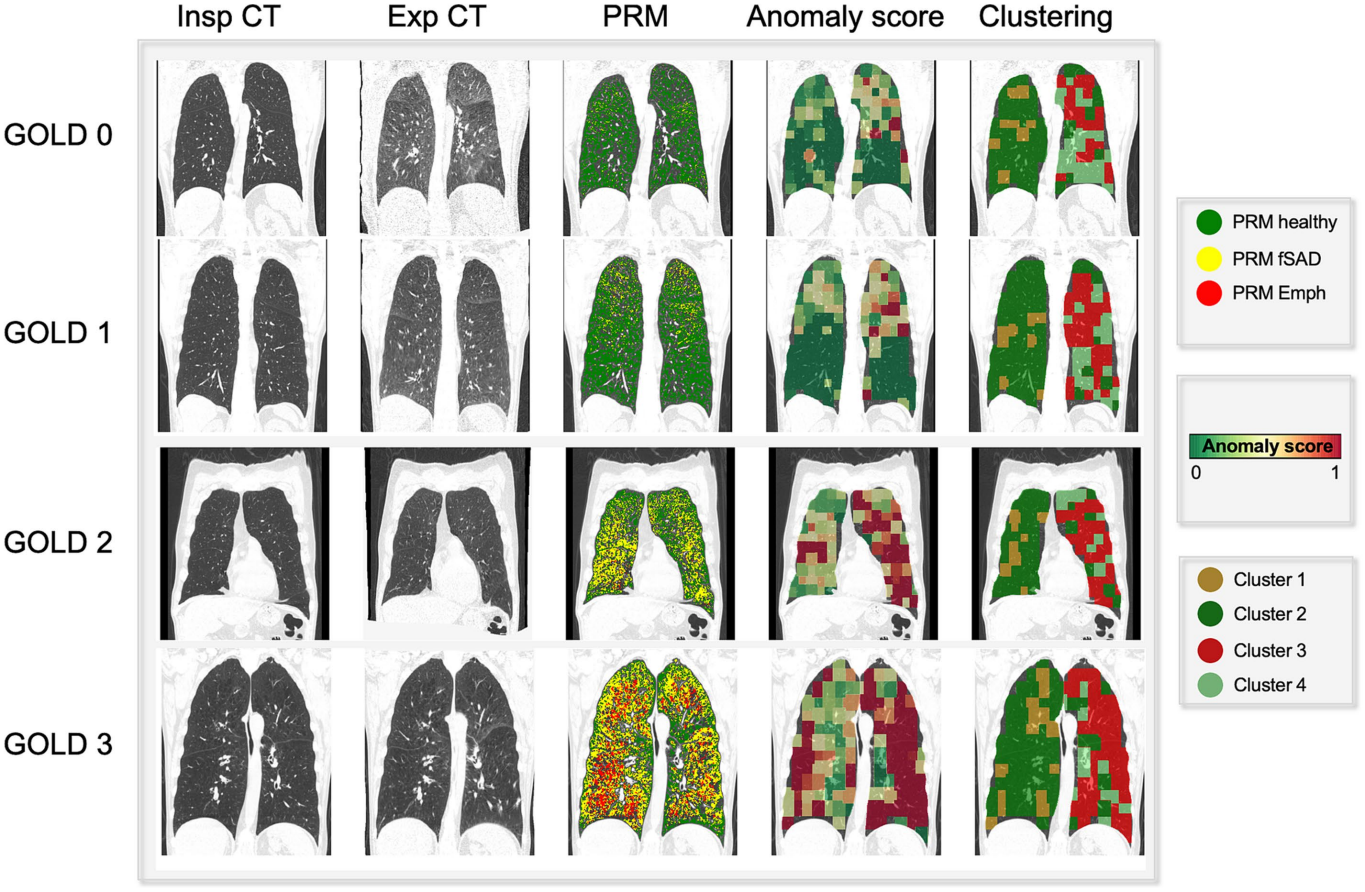
Visual comparison of PRM maps, anomaly score and cluster volumes on exemplary subjects across several GOLD stages (0–4). As the severity increases (GOLD score), so do the areas detected as emphysema and functional small airway disease by PRM. On the same fashion, the region anomaly scores also shift from green to red for more severe cases. Interestingly, these areas overlap PRM^Emph^ and PRM^fSAD^. Particularly for low GOLD levels, it attributes higher scores to PRM^fSAD^ areas, possibly indicative of differential progressive features. Lastly, the cluster maps reveal four aggregations, indicative of different disease components.

**Figure 4:**
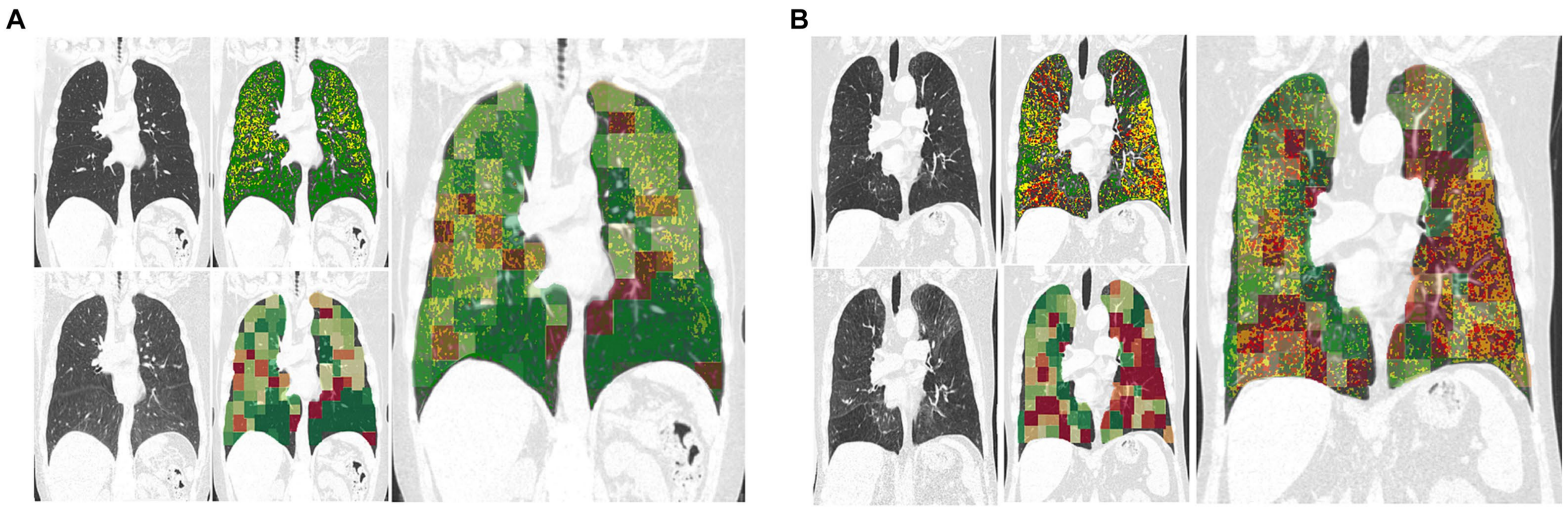
Exemplary coronal sections of overlapping of PRM and anomaly maps for two former-smoker individuals. Example **(A)** is a 64 years-old former-smoker female who does not fulfill the criteria for COPD (GOLD 0; FEV1% post-bronchodilator 126% of predicted value; FEV1/FVC 78%; smoking history of 30 years). Example **(B)** is a 72 years-old former-smoker male with severe COPD (GOLD 3; FEV1% post-bronchodilator 31% of predicted value; FEV1/FVC 34%; smoking history of 37 years). Top left corresponds to the inspiratory scans, top right to the PRM maps, bottom left to the expiratory registered scan, bottom right to the anomaly score map. The bigger image corresponds to the overlapping of the anomaly maps to the PRM maps, in which regions deviating from the norm are overlapping with PRM^fSAD^. The closer to red, the higher the anomaly detected. PRM healthy tissue (green), PRM functional small-airway disease (yellow), and PRM emphysema (red).

Figure 3 showcases representative CT images individuals from all GOLD grades. The first and second column represent a coronal slice of the inspiratory and expiratory CT scans, while the following columns are the visualization of PRM maps, maps of the anomaly score and cluster classes, overlayed on the inspiratory CT. Figure 4 represents the spatial overlap between region-wise anomaly scores and PRM^Emph^ and PRM^fSAD^ for two former-smoker individuals: GOLD 0 (A) and GOLD 3 (B), with more than 30 years of smoking history.

### 3.4 PRM volumes, anomaly score, cluster groups by GOLD score

**Figure 5:**
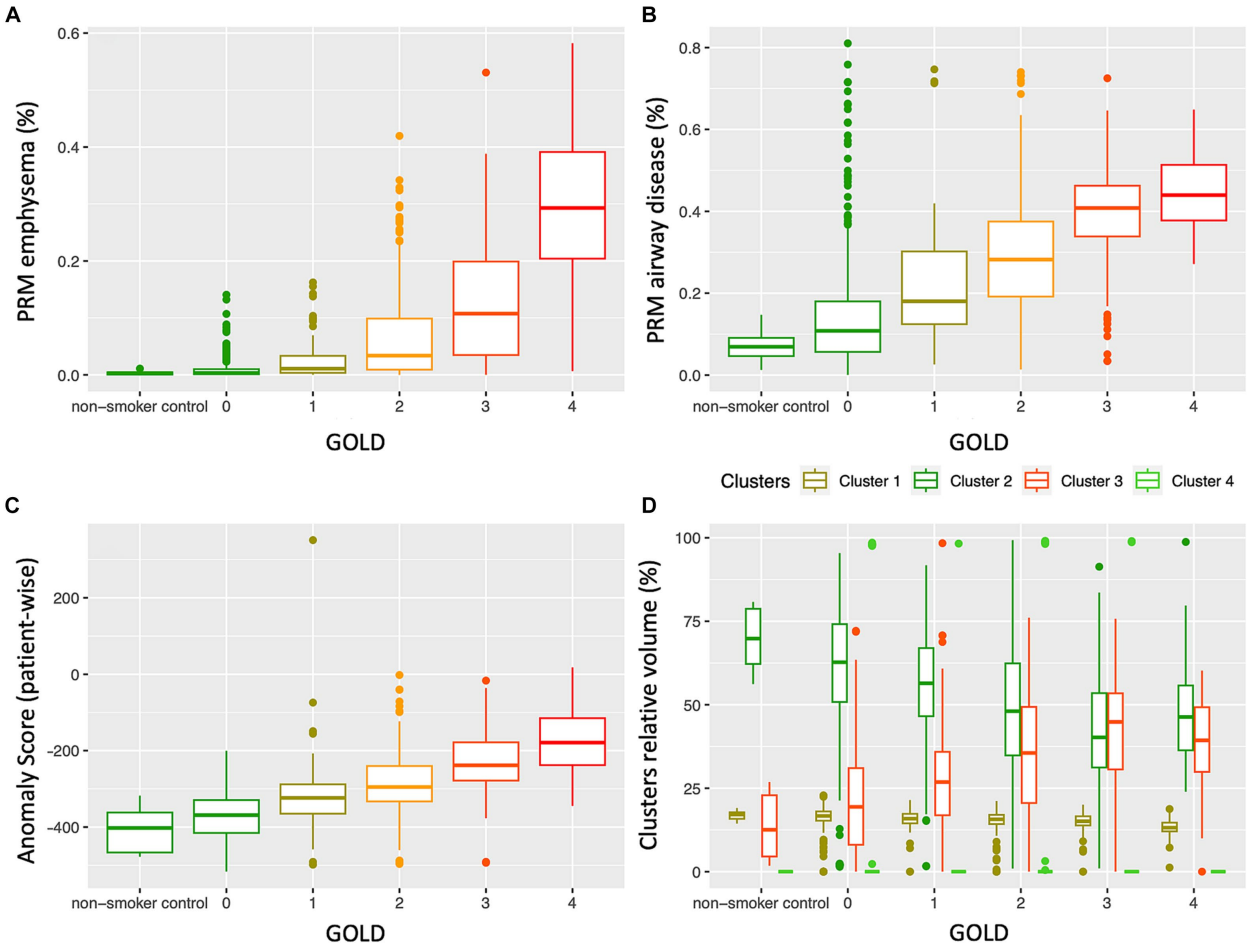
Distribution of PRM emphysema **(A)**, PRM airway disease (fSAD) **(B)**, patient-wise anomaly score **(C)** and cluster classes **(D)**, according to the GOLD stage. While PRM^Emph^, PRM^fSAD^ and the anomaly score clearly increase with the disease severity, the relative volumes of the cluster classes showed distinctly different distributions according to GOLD severity; cluster 3 increases, whereas cluster 2 decreases with an increase in GOLD stage. GOLD, Global Initiative for Obstructive Lung Disease.

Figure 5 depicts a comparison between PRM volumes, anomaly score and relative size of the 4 clusters, relatively to the GOLD stage. Significant differences were found between PRM volumes, anomaly scores and all cluster classes according to the GOLD stage (Table 2), except for class 4. With an increase in the GOLD stage, PRM^Emph^, PRM^fSAD^, Anomaly Score and relative volume of cluster 3 increased, while cluster 2 decreased. Post-hoc Tukey analysis (Supplementary Table S1) revealed significant intergroup differences in the anomaly score between all GOLD stages (*p* < 0.01 corrected), except between controls and GOLD 0. No differences were observed between controls and GOLD 0, controls and GOLD 1, and GOLD 0 and GOLD 1 for PRM^Emph^; and controls and GOLD 0 and GOLD 4 and GOLD 3 for PRM^fSAD^. Cluster 1 showed significant differences between high grades COPD (GOLD 3 and 4), and Cluster 2 between GOLD 2 and 3. Notably, statistically significant differences between never-smoker controls and GOLD 0 subjects were only found by Cluster 3.

**Table 2:** Mean ± standard deviation of parametric response mapping (PRM) volumes, anomaly score and cluster groups, according to the GOLD stage.

	Controls (*N* = 29)	GOLD 0 (*N* = 538)	GOLD 1 (*N* = 128)	GOLD 2 (*N* = 315)	GOLD 3 (*N* = 195)	GOLD 4 (*N* = 105)	*p*-value
PRM
Healthy (%)	92.6 ± 4.1	84.4 ± 14.8	75.4 ± 16.2*	64.2 ± 19.3*	47.3 ± 18.4*	25.4 ± 10.3*	<0.001
Functional small-airway (%)	7.1 ± 4.0	14.7 ± 13.8	22.0 ± 13.8*	28.9 ± 14.0*	39.4 ± 11.5*	45.1 ± 8.5	<0.001
Emphysema (%)	0.3 ± 0.3	0.9 ± 1.7	2.6 ± 3.6	6.8 ± 8.0*	13.3 ± 11.3*	29.5 ± 13.6*	<0.001
Anomaly detection (cOOpD)							
Anomaly score	−404.1 ± 54.3	−372.0 ± 63.8	−318.9 ± 100.8*	−287.8 ± 80.8*	−228.0 ± 77.4*	−170.9 ± 82.2*	<0.001
Clusters							
1	16.8 ± 1.4	16.2 ± 3.4	15.5 ± 3.2	15.3 ± 3.3	14.9 ± 2.7	13.2 ± 2.7*	<0.001
2	69.7 ± 9.0	60.7 ± 17.8	55.7 ± 17.7	48.9 ± 19.4	42.2 ± 16.4*	48.5 ± 15.1	<0.001
3	13.5 ± 9.3	21.1 ± 15.7*	27.7 ± 17.4	34.2 ± 18.5	41.7 ± 16.3*	38.2 ± 13.8	<0.001
4	0.0 ± 0.0	1.9 ± 13.6	1.1 ± 10.2	1.6 ± 12.5	1.2 ± 10.8	0.0 ± 0.0	n.s.

GOLD, Global Initiative for Obstructive Lung Disease. *p-*value was obtained by the Jonckheere-Terpstra test. * denotes *p* < 0.05 vs. previous GOLD stage for the post-hoc Tukey-test comparison.

### 3.5 Relationships between PRM volumes, anomaly score, cluster groups

Table 3 shows the Pearson correlation coefficient and *p*-values corrected with Holm-Bonferroni for PRM volumes, anomaly score and cluster groups. Each PRM volume showed statistically significant correlations with PFTs and clinical data. Both PRM^fSAD^, PRM^Emph^, Anomaly score and Cluster 3 are negatively correlated with spirometry volumes (FEV1 and FEV1/FVC) and with the walking distance, and positively correlated with FRC, TLC, FRC/TLC, BODE and smoking duration. Pearson correlation coefficients for the anomaly score are moderate, with the exception of FRC and smoking duration, and very well comparable with the ones from PRM volumes, while less strong for the Cluster 3. The anomaly score showed significantly higher correlations than PRM^fSAD^ for SGRQ and the distance walked in the 6-min walking test; and stronger than PRM^Emph^ for FRC/TLC and smoking duration. No significant differences were found between correlations of the anomaly score and PRM^fSAD^ for FEV1, FEV1/FVC, BODE and smoking duration; and between the anomaly score and PRM^Emph^ for FEV1, FRC, SGRQ and the distance walked in the 6-min walking test. The anomaly scores also showed significantly higher correlations than all Clusters for all clinical variables, except for TLC. Clusters 1 and 2 follow the same trends as the PRM Healthy volumes: positively correlated with FEV1, FEV1/FVC and walking distances and negatively correlated with FRC, TLC, FRC/TLC, BODE, SGRQ, and smoking duration.

**Table 3:** Correlation of PRM volumes, anomaly score, and cluster groups with PFTs and clinical data.

	Method	Pulmonary function tests and clinical data								
		FEV1	FEV1/FVC	TLC	FRC	FRC/TLC	BODE	SGRQ	6MWT	Duration smoking
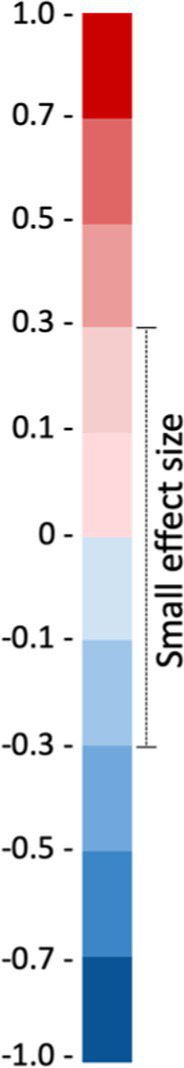	PRM									
	Healthy (%)	0.71**** (0.68, 0.74)	0.80**** (0.77, 0.82)	−0.30**** (−0.35, −0.24)	−0.72**** (−0.75, −0.69)	−0.73**** (−0.76, −0.70)	−0.64**** (−0.68, −0.60)	−0.46**** (−0.51, −0.41)	0.42**** (0.37, 0.47)	−0.27**** (−0.30, −0.20)
	PRM^fSAD^ %	−0.61**** (−0.66, −0.57)	−0.68**** (−0.72, −0.64)	0.24**** (0.18, 0.30)	0.70**** (0.67, 0.73)	0.78**** (0.76, 0.81)	0.53**** (0.48, 0.57)	0.39**** (0.34, 0.44)	−0.34**** (−0.39, −0.30)	0.28**** (0.20, 0.31)
	PRM^Emph^ %	−0.66**** (−0.69, −0.63)	−0.75**** (−0.77, −0.72)	0.30**** (0.24, 0.36)	0.56**** (0.51, 0.61)	0.44**** (0.39, 0.50)	0.64**** (0.60, 0.68)	0.43**** (0.38, 0.48)	−0.42**** (−0.47, −0.37)	0.18**** (0.11, 0.21)
	Anomaly detection (cOOpD)									
	Anomaly score	−0.65**** (−0.69, −0.60)	−0.68**** (−0.72, −0.64)	0.16**** (0.09, 0.22)	0.55**** (0.51, 0.60)	0.56**** (0.51, 0.62)	0.57**** (0.52, 0.62)	0.46**** (0.41, 0.51)	−0.41**** (−0.47, −0.35)	0.27**** (0.19, 0.30)
	Clusters									
	1	0.22**** (0.17, 0.29)	0.27**** (0.21, 0.34)	−0.22**** (−0.28, −0.15)	−0.34**** (−0.41, −0.27)	−0.35**** (−0.43, −0.27)	−0.23**** (−0.29, −0.16)	−0.15**** (−0.21, −0.09)	0.16**** (0.10, 0.22)	−0.10*** (−0.15, −0.03)
	2	0.33**** (0.28, 0.38)	0.34**** (0.28, 0.39)	−0.06* (−0.12, −0.01)	−0.22**** (−0.28, −0.16)	−0.17**** (−0.25, −0.09)	−0.25**** (−0.30, −0.19)	−0.21**** (−0.26, −0.15)	0.20**** (0.14, 0.26)	−0.16**** (−0.19, −0.07)
	3	−0.41**** (−0.46, −0.36)	−0.42**** (−0.47, −0.37)	0.11*** (0.05, 0.17)	0.32**** (0.27, 0.38)	0.29**** (0.22, 0.36)	0.32**** (0.27, 0.38)	0.27**** (0.22, 0.32)	−0.24**** (−0.30, −0.19)	0.18**** (0.10, 0.21)
	4	0.04 (−0.01, 0.10)	0.04 (−0.02, 0.09)	−0.01 (−0.06, 0.04)	−0.06 (−0.10, −0.02)	−0.08* (−0.11, −0.05)	−0.04 (−0.10, 0.02)	−0.04 (−0.10, 0.01)	0.00 (−0.05, 0.06)	0.00 (−0.07, 0.04)

Confidence intervals are denoted in brackets, per each Pearson correlation coefficient. Correlations are colored according to Cohen’s effect size (26) and only the significant ones are colored. None or very weak effect size is defined by |*r*| < 0.3, weak 0.3 < |*r*| <0.5, moderate 0.5 < |*r*| < 0.7 and large by |*r*| > 0.7. PRM, parametric response mapping; fSAD, functional Small Airway Disease; Emph, Emphysema; FEV1, Forced Expiratory Volume in 1 s, FEV1/FVC, FEV/Forced Vital Capacity; FRC, Functional Residual Capacity; TLC, Total Lung Capacity; BODE, Body mass index, air-flow Obstruction, Dyspnea, Exercise capacity; SGRQ, St George’s Respiratory Questionnaire; 6MWT, 6 min walking test. **** *p* < 0.0001, *** *p* < 0.001, ** *p* < 0.01, *p < 0.05.

**Figure 6:**
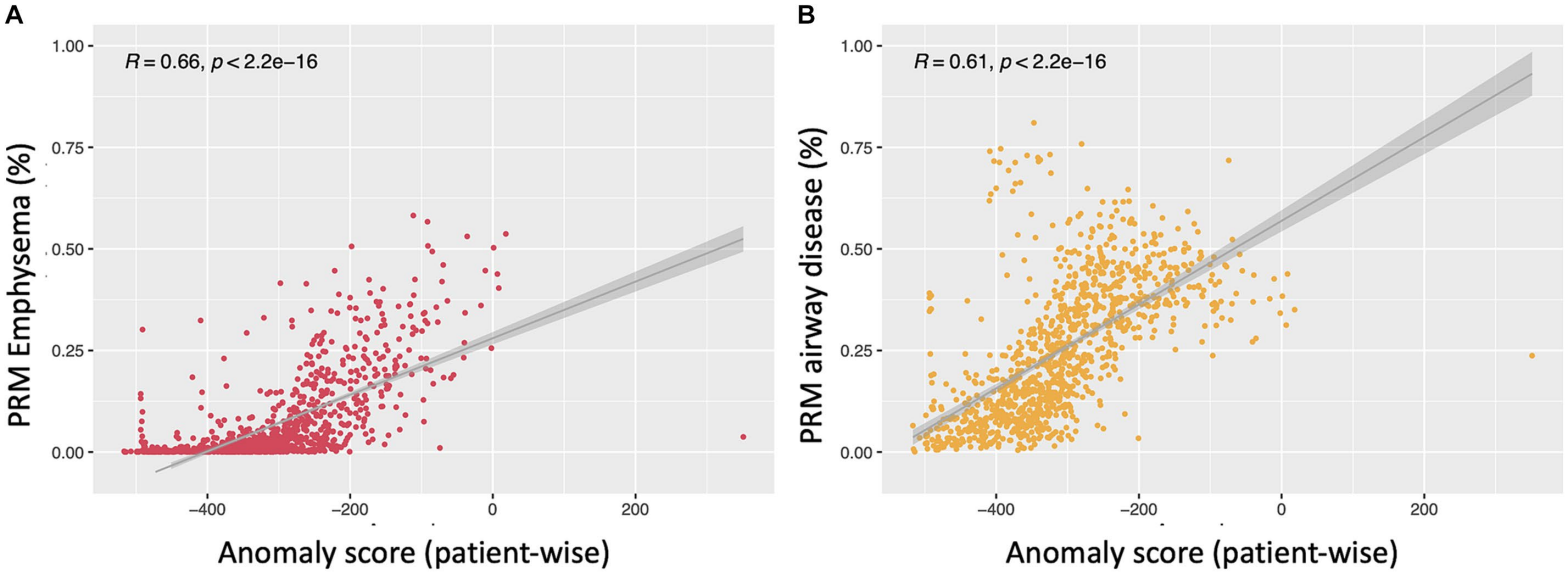
Scatterplots depicting the relationship between the anomaly score and PRM emphysema **(A)** and PRM^fSAD^ **(B)**. Both show linear regression between patient-wise anomaly score and PRM volumes in all subjects (PRM^Emph^: *r* = 0.66, *p* < 0.01; PRM^fSAD^: *r* = 0.61 *p* < 0.01).

Figure 6 illustrates linear relationships between patient-wise anomaly scores and PRM-derived emphysema and fSAD volumes. The anomaly score shows to be significantly correlated with PRM^Emph^ (*r* = 0.66, *p* < 0.01) and PRM^fSAD^ (*r* = 0.61 *p* < 0.01). As depicted in Table 4, adding the anomaly score to the PRM baseline models statistically improves the fit of all LMM to predict clinical measures, including FEV1, FEV/FVC, FRC, FRC/TLC, BODE, SGRQ, and 6MWT (only to PRM^fSAD^). No differences were found for predicting the TLC and the 6MWT (for PRM^Emph^).

**Table 4:** Linear mixed effects models were employed to predict several clinical variables including FEV1%, FEV/FVC, FRC, TLC, FRC/TLC, BODE, SGRQ, and 6MWT, with adjustments made for age, gender, BMI, smoking status, smoking duration, and study site (baseline models).

Dependent variable	Predictor	Adjusted conditional *R*^2^	*p*-value adjusted
FEV1%	Age, gender, BMI, smoking status, smoking duration (center)	0.22	
	Age, gender, BMI, smoking status, smoking duration, **PRM^fSAD^** (center)	0.46	*p* < 0.001
	Age, gender, BMI, smoking status, smoking duration, **PRM^fSAD^, anomaly score** (center)	**0.56**	
	Age, gender, BMI, smoking status, smoking duration, **PRM^Emph^** (center)	0.53	*p* < 0.001
	Age, gender, BMI, smoking status, smoking duration, **PRM^Emph^, anomaly score** (center)	**0.58**	
FEV1/FVC	Age, gender, BMI, smoking status, smoking duration (center)	0.26	
	Age, gender, BMI, smoking status, smoking duration, **PRM^fSAD^** (center)	0.54	*p* < 0.001
	Age, gender, BMI, smoking status, smoking duration, **PRM^fSAD^, anomaly score** (center)	**0.63**	
	Age, gender, BMI, smoking status, smoking duration, **PRM^Emph^** (center)	0.63	*p* < 0.001
	Age, gender, BMI, smoking status, smoking duration, **PRM^Emph^, anomaly score** (center)	**0.66**	
TLC	Age, gender, BMI, smoking status, smoking duration (center)	0.50	
	Age, gender, BMI, smoking status, smoking duration, **PRM^fSAD^** (center)	0.54	n.s
	Age, gender, BMI, smoking status, smoking duration, **PRM^fSAD^, anomaly score** (center)	0.55	
	Age, gender, BMI, smoking status, smoking duration, **PRM^Emph^** (center)	0.56	n.s
	Age, gender, BMI, smoking status, smoking duration, **PRM^Emph^, anomaly score** (center)	0.56	
FRC	Age, gender, BMI, smoking status, smoking duration (center)	0.32	
	Age, gender, BMI, smoking status, smoking duration, **PRM^fSAD^** (center)	0.67	*p* < 0.001
	Age, gender, BMI, smoking status, smoking duration, **PRM^fSAD^, anomaly score** (center)	**0.69**	
	Age, gender, BMI, smoking status, smoking duration, **PRM^Emph^** (center)	0.54	*p* < 0.001
	Age, gender, BMI, smoking status, smoking duration, **PRM^Emph^, anomaly score** (center)	**0.58**	
FRC/TLC	Age, gender, BMI, smoking status, smoking duration (center)	0.23	
	Age, gender, BMI, smoking status, smoking duration, **PRM^fSAD^** (center)	0.68	*p* < 0.001
	Age, gender, BMI, smoking status, smoking duration, **PRM^fSAD^, anomaly score** (center)	**0.69**	
	Age, gender, BMI, smoking status, smoking duration, **PRM^Emph^** (center)	0.36	*p* < 0.001
	Age, gender, BMI, smoking status, smoking duration, **PRM^Emph^, anomaly score** (center)	**0.41**	
BODE	Age, gender, BMI, smoking status, smoking duration (center)	0.21	
	Age, gender, BMI, smoking status, smoking duration, **PRM^fSAD^** (center)	0.37	*p* < 0.001
	Age, gender, BMI, smoking status, smoking duration, **PRM^fSAD^, anomaly score** (center)	**0.45**	
	Age, gender, BMI, smoking status, smoking duration, **PRM^Emph^** (center)	0.49	*p* < 0.001
	Age, gender, BMI, smoking status, smoking duration, **PRM^Emph^, anomaly score** (center)	**0.51**	
SGRQ	Age, gender, BMI, smoking status, smoking duration (center)	0.24	
	Age, gender, BMI, smoking status, smoking duration, **PRM^fSAD^** (center)	0.35	*p* < 0.001
	Age, gender, BMI, smoking status, smoking duration, **PRM^fSAD^, anomaly score** (center)	**0.41**	
	Age, gender, BMI, smoking status, smoking duration, **PRM^Emph^** (center)	0.39	*p* < 0.001
	Age, gender, BMI, smoking status, smoking duration, **PRM^Emph^, anomaly score** (center)	**0.42**	
6MWT	Age, gender, BMI, smoking status, smoking duration (center)	0.41	
	Age, gender, BMI, smoking status, smoking duration, **PRM^fSAD^** (center)	0.42	*p* < 0.001
	Age, gender, BMI, smoking status, smoking duration, **PRM^fSAD^, anomaly score** (center)	**0.45**	
	Age, gender, BMI, smoking status, smoking duration, **PRM^Emph^** (center)	0.48	n.s.
	Age, gender, BMI, smoking status, smoking duration, **PRM^Emph^, anomaly score** (center)	0.48	

PRM volumes (PRM^Emph^ and PRM^fSAD^) were then incorporated as predictors. Subsequently, these models were compared to those further adjusted for the anomaly score. Conditional *R*^2^ values are adjusted for the number of regressors added. Bold values indicate a higher *R*^2^ for each dependent variable. All models were statistically significant compared to the baseline model. *p*-values are reported for the comparison between PRM baseline models and anomaly-adjusted PRM models, and are corrected for multiple comparisons.

**Figure 7:**
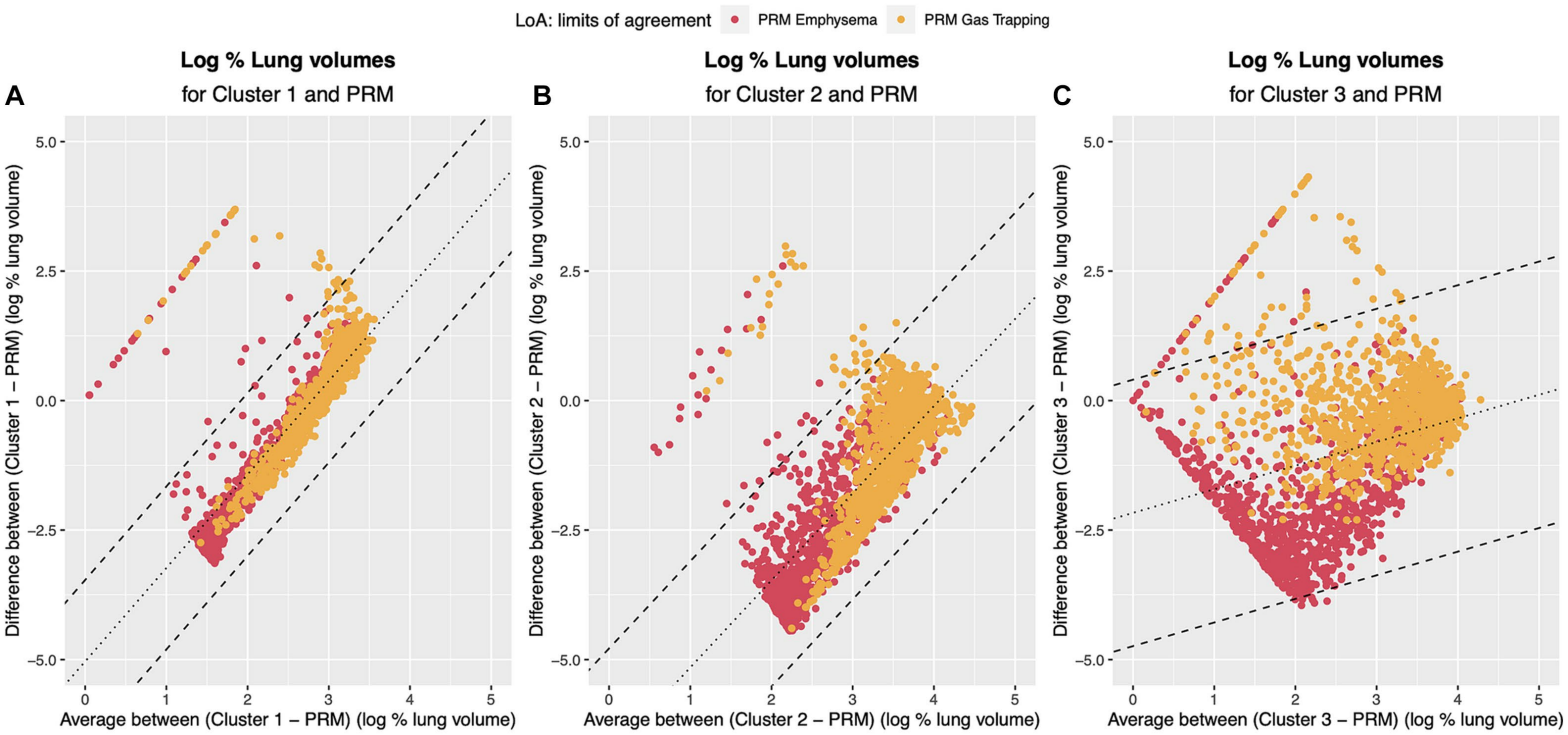
Bland–Altman plots regression-based limits of agreement. Dotted line represents the mean difference between the methods **(A)** PRM and Cluster 1: *Dˆ_PRM,Cluster1_*=−5.04 + 1.80 *A*; **(B)** PRM and Cluster 2 *Dˆ_PRM,Cluster2_*=−6.84 + 1.68 *A*; **(C)** PRM and Cluster 3 *Dˆ_PRM,Cluster3_*=−2.17 + 0.46 *A*. Dashed line represents the 95% Limits of Agreement: **(A)** PRM and Cluster 1: *Dˆ_PRM,Cluster1_*=−5.04 + 1.80 *A* ± 0.80; **(B)** PRM and Cluster 2 *Dˆ_PRM,Cluster2_*=−6.84 + 1.68 *A* ± 1.05; **(C)** PRM and Cluster 3 *Dˆ_PRM,Cluster3_*=−2.17 + 0.46 *A* ± 1.31. Red points refer to the agreement to PRM emphysema and yellow to the agreement to PRM small airway disease.

Bland–Altman plots for the volume (log transformed) agreement of PRM-derived volumes and clusters which showed significant correlation to the PFTs and clinical data (Clusters 1, 2, 3) are presented in Figure 7 showing the differences (D) against averages (A). Since the Bland–Altman plots displayed non-constant bias even after log transformation of the measurements, a regression-based approach (25) was used to compute the bias and limits of agreement (LoA). The mean differences are given by ${\hat{D}}_{PRM,\mathrm{Cluster}\;1}=-5.04+1.80\;A$ , ${\hat{D}}_{PRM,\mathrm{Cluster}\;2}=-6.84+1.68\;A$ , ${\hat{D}}_{PRM,\mathrm{Cluster}\;3}=-2.17+0.46\;A$ . The 95% LoA are then $\begin{array}{l} {\hat{D}}_{PRM,\mathrm{Cluster}\;1}=-5.04+1.80\;A\\ \;\pm 0.80 \end{array}$ , $\begin{array}{l} {\hat{D}}_{PRM,\mathrm{Cluster}\;2}=-6.84+1.68\;A\\ \;\pm 1.05 \end{array}$ , $\begin{array}{l} {\hat{D}}_{PRM,\mathrm{Cluster}\;3}=-2.17+0.46\;A\\ \;\pm 1.31 \end{array}$ . Thus, the degree of agreement is not constant across the range of measurements.

## 4 Discussion

In this study, we aimed to explore the potential of a deep-learning self-supervised anomaly detection method for phenotyping Chronic Obstructive Pulmonary Disease (COPD) using computed tomography (CT) scans of 1,310 never-smoker controls and GOLD 0–4 from the COPDGene cohort. COPD remains a significant global health burden, necessitating early detection and accurate phenotyping for effective intervention. While traditional methods like parametric response mapping (PRM) have provided insights into COPD pathophysiology (27, 28), they may not fully capture the complexity and heterogeneity of the disease. This present study marks the first of its kind, comparing PRM-derived functional small-airway disease and emphysema measurements against regional anomaly scores derived from a recently proposed self-supervised deep-learning anomaly detection approach.

Before delving into the comparison itself, it was important to understand what spatial distribution is encoded by the self-supervised latent features. As unveiled through t-distributed Stochastic Neighbor Embedding (t-SNE) analysis, it showcases a notable attraction among similar disease-related areas, concurrently distancing healthy regions from those corresponding to healthy patients. Notably, this visualization is rooted in the self-supervised contrastive learning of lung regions and, as such, does not rely on labels. It is noteworthy that this approach effectively captures features stemming from emphysema and gas trapping, while factors such as gender information do not contribute to this attraction. Furthermore, an intriguing observation surfaces in COPD patients, where even regions with low levels of emphysema exhibit spatial resemblances with areas demonstrating higher emphysema levels. This pattern potentially hints at underlying features beyond emphysema that play a role in clustering these regions together, possibly indicating the presence of early-stage COPD markers that could progress into advanced emphysematous changes.

Investigating the spatial relationships between small airway disease and emphysema manifestations captured by PRM alongside regions identified as anomalies by the self-supervised approach has revealed interesting insights. Traditionally, the progression of COPD involves the narrowing or destruction of small airways preceding the development of emphysema (29), a transition not easily detectable using conventional spirometry or visible on CT scans (27, 30). Our exemplary patients’ regions, colored solely by PRM^fSAD^, often exhibit areas with higher anomaly scores in the anomaly maps, as illustrated in Figures 3, 4A. This observation raises the intriguing possibility that the anomaly detection approach serves as an additional means to highlight transitional stages in the disease process, paramount for understanding early pathological changes (31). It may also suggest that small airway disease undergoes dynamic changes before evolving into more advanced stages of emphysema, particularly relevant for individuals with normal lung function (GOLD 0, Figure 4A). Therefore, more importantly than the spatial similarity, the divergence observed between anomaly maps and PRM classes indicates the potential of the anomaly detection method to detect early-stage disease manifestations not yet captured by traditional phenotyping methods, particularly in regions of individuals with a long smoking history (Figure 4). Further investigations, including the assessment of normal CT voxels alongside CT airway measurements and PRM analyses, are warranted to elucidate the underlying pathophysiological mechanisms responsible for these observations and to validate these findings in prospective longitudinal studies. Furthermore, our study revealed a significant positive correlation between the patient-wise anomaly scores and PRM-derived fSAD (*r* = 0.61, *p* < 0.01) and Emphysema volumes (*r* = 0.66, *p* < 0.01). Overall, these findings underscore the complementary nature of the two approaches and the potential benefit of integrating them to achieve a more comprehensive assessment of COPD heterogeneity.

Moreover, our study reveals a parallel finding to Hwang HJ et al. (32), where a novel emphysema air-trapping composite (EAtC) has been proposed. In particular, their functional air trapping (fAT) component captures air trapping in both emphysematous and non-emphysematous areas. Just as in their work, our investigation suggests that employing the anomaly detection comprehensive approach yields better or comparable correlations with clinical variables (SGRQ, 6MWT; =FEV1, =FEV1/FVC, =BODE and = smoking duration) compared to conventional PRM-based small-airway disease assessments, which focus solely on non-emphysematous air trapping. Similarly, the anomaly score exhibited stronger correlations than PRM^Emph^ for parameters like FRC/TLC and smoking duration, while no differences were found for FEV1, FRC, SGRQ and the distance walked in the 6-min walking test. This suggests the ability of the anomaly detection approach in characterizing small-airway disease and emphysema more comprehensively, potentially encompassing aspects that conventional methods might overlook. Subsequent analysis confirmed this hypothesis, as adding the anomaly score to LMM adjusted for both PRM volumes, significantly improved the prediction of clinical variables (FEV1, FEV/FVC, FRC, FRC/TLC, BODE, SGRQ). This provides further evidence that the anomaly score captures nuanced features beyond the structural characteristics from the PRM analysis and underscores their complementary nature.

While the debate surrounding air trapping in emphysematous regions remains, evidence from both Hwang HJ et al. (32) and our study supports the notion that small-airway disease is not confined solely to areas with preserved alveoli but can coexist within emphysematous zones as well. This duality emphasizes the interplay between different disease manifestations and highlights the potential for anomalies, as captured by the anomaly detection approach, to encompass both emphysematous and non-emphysematous regions. Additionally, it may also explain why, in contrast to PRM^fSAD^ and PRM^Emph^, the patient-wise anomaly score significantly differs across all GOLD stages (*p* < 0.01), except between never-smoker controls and GOLD 0.

Our cluster analysis and visualization of PRM-derived regions have illuminated distinct patterns within the dataset. By employing principal component analysis (PCA) for dimensionality reduction, we successfully identified four stable clusters that exhibited variations corresponding to different stages of the disease. Notably, Cluster 1 appears to embody regions that persist consistently across all GOLD stages, indicative of common characteristics shared across disease phenotypes. Cluster 2, on the other hand, emerges as a distinct subset that represents healthy regions and unaffected by the disease, potentially explaining its volume decline as disease severity increases. The most intriguing finding lies in Cluster 3, where regions manifest features closely aligned with PRM-derived fSAD (Figure 7), which suggests an association with early-stage COPD characteristics. It is conceivable that Cluster 1 represents areas that have never experienced the impact of COPD, while Cluster 3 may signify regions embarking on the initial stages of disease progression. This interpretation finds support in the progressively increasing representation of Cluster 3 across GOLD classes, hinting at its role in the evolution of the disease from milder to more advanced stages. Although the correlations observed between PFTs and PRM-derived Emphysema and fSAD classes are superior to Cluster 3, Cluster 3 is the only able to differentiate never-smoker controls and GOLD 0 subjects. Amidst these notable clusters, Cluster 4 adds a layer of complexity. While present in a smaller subset of patients (*n* = 24), notably it covers over 50% of the lung in 22 individuals, showing distinct clinical features like high gas trapping (31.3 ± 23.4%) and extensive smoking history (33 ± 12 years). Still, the decision to retain it was supported by robust clustering metrics detailed in the Supplementary materials.

The non-constant bias observed in the Bland–Altman analysis suggests that the agreement between PRM-derived volumes and cluster volumes is not uniform across the entire range of measurements. This non-uniformity may stem from inherent differences in how PRM and the clustering method characterize and quantify lung regions. The regression-based equations provide a nuanced understanding of this non-constant bias, indicating that the discrepancy between methods is influenced by the magnitude of the measurements. These findings may imply that the clusters, despite their ability to capture distinct patterns related to COPD stages, may not uniformly agree with PRM-derived volumes across all levels of disease severity. The varying agreement observed in different parts of the measurement range could be attributed to the complex and heterogeneous nature of COPD, where different phenotypes and disease manifestations may impact the agreement between methods differently. Moreover, it’s important to acknowledge an unexpected discrepancy observed in the distribution of clusters between the left and right lungs. Visual inspection of the original inspiratory and expiratory CT scans did not consistently support the distinct left/right lung asymmetry detected by the clustering algorithm. Notably, we observed an apparent concentration of clusters 3 and 4 in the left lung and clusters 1 and 2 in the right lung. This left/right lung asymmetry prompts caution in the interpretation of cluster-specific findings. While our primary objectives center around the comparative analysis between PRM and anomaly detection, this unexpected observation underscores the need for transparency regarding potential sources of bias in the clustering component. This information adds a layer of transparency to our study, acknowledging that the agreement between PRM and clustering might be influenced by factors that vary across different regions of the lung or disease states. Importantly, it’s worth noting that the anomaly score, which consistently demonstrated significant correlations with PRM-derived measurements and clinical data, exhibited a more uniform agreement, providing a robust and complementary perspective on COPD phenotyping. Our findings also indicate that the anomaly detection method provides a more robust and complementary perspective on COPD phenotyping compared to Clustering. This is demonstrated by statistically significant higher correlations between anomaly scores and clinical variables, along with larger effect sizes. Anomaly detection’s ability to identify deviations from the distribution of “normal” samples makes it better suited for capturing subtle variations indicative of disease pathology, contrasting with Clustering’s reliance on proximity-based grouping, while based on the exact same latent features.

Furthermore, while our findings indicate a strong potential for anomaly detection in COPD phenotyping, further research is needed to establish its generalizability across diverse populations. Additionally, our current exploration does not delve into distinguishing COPD from other comorbidities, such as lung cancer, warranting careful consideration and validation in future investigations.

In conclusion, we introduce the anomaly detection approach as a novel perspective in COPD phenotyping, extending the lens beyond threshold-based analysis and methodologies focused on distinct imaging features. By identifying anomalies spanning a spectrum of disease features, including those beyond functional small-airway disease and emphysema, we unveil a more comprehensive viewpoint, offering an additional layer of information that goes beyond traditional PRM phenotyping. The resulting insights offer a window into the intricate distribution of the disease. This transparency in AI diagnostics empowers clinicians to personalize interventions while supplementing established methods. By integrating this innovative approach into clinical practice, we lay the groundwork for refined diagnostics and tailored interventions, ultimately leading to enhanced patient outcomes.

## Data availability statement

The original contributions presented in the study are included in the article/Supplementary material, further inquiries can be directed to the corresponding authors.

## Ethics statement

Ethics approval conducted in COPDGene. The studies were conducted in accordance with the local legislation and institutional requirements. The participants provided their written informed consent to participate in this study.

## Author contributions

SA: Conceptualization, Data curation, Formal analysis, Investigation, Methodology, Validation, Visualization, Writing – original draft, Writing – review & editing. TN: Funding acquisition, Methodology, Project administration, Supervision, Writing – original draft, Writing – review & editing. CL: Investigation, Methodology, Writing – original draft, Writing – review & editing, Validation. TW: Investigation, Methodology, Writing – original draft, Writing – review & editing. VW: Data curation, Formal analysis, Supervision, Validation, Visualization, Writing – original draft, Writing – review & editing. MN: Supervision, Writing – original draft, Writing – review & editing. PJ: Supervision, Writing – original draft, Writing – review & editing. OS: Funding acquisition, Project administration, Writing – original draft, Writing – review & editing. CH: Investigation, Supervision, Writing – original draft, Writing – review & editing. OW: Investigation, Supervision, Writing – original draft, Writing – review & editing. JB: Investigation, Supervision, Validation, Visualization, Writing – original draft, Writing – review & editing. H-UK: Writing – original draft, Writing – review & editing. KM-H: Conceptualization, Supervision, Writing – original draft, Writing – review & editing.
