# Supplementary material for: Capturing COPD heterogeneity: anomaly detection and parametric response mapping comparison for phenotyping on chest computed tomography
Source: Front Med (Lausanne). 2024 Mar 1;11:1360706. doi: 10.3389/fmed.2024.1360706 (PMC10941845; doi:10.3389/fmed.2024.1360706)
Supplement: Supplementary file 1 [file Data_Sheet_1.docx]

Supplementary Material

# Principal Component Analysis on the self-supervised latent features

Principal Component Analysis (PCA) was applied as a further dimensionality reduction technique on the 1x512 feature vector, corresponding to a latent feature from each 3D patch (50 x 50 x 50) of the lung parenchyma.

First, the first eigenvalues were visualized (Supplementary Figure 1), to show the percentage of variances explained by each principal component. Then, Horn’s parallel analysis was applied to assess the number of components to keep in the PCA analysis. This analysis is based on the assumption that if the data was random, non-correlated factors would be observed and the eigenvalue of PCA would be 1, i.e. to compare the obtained PCA results with random data PCA results. After excluding the proportion of variance resulting from sampling error (Supplementary Figure 2), 85 PCs were retained.

*
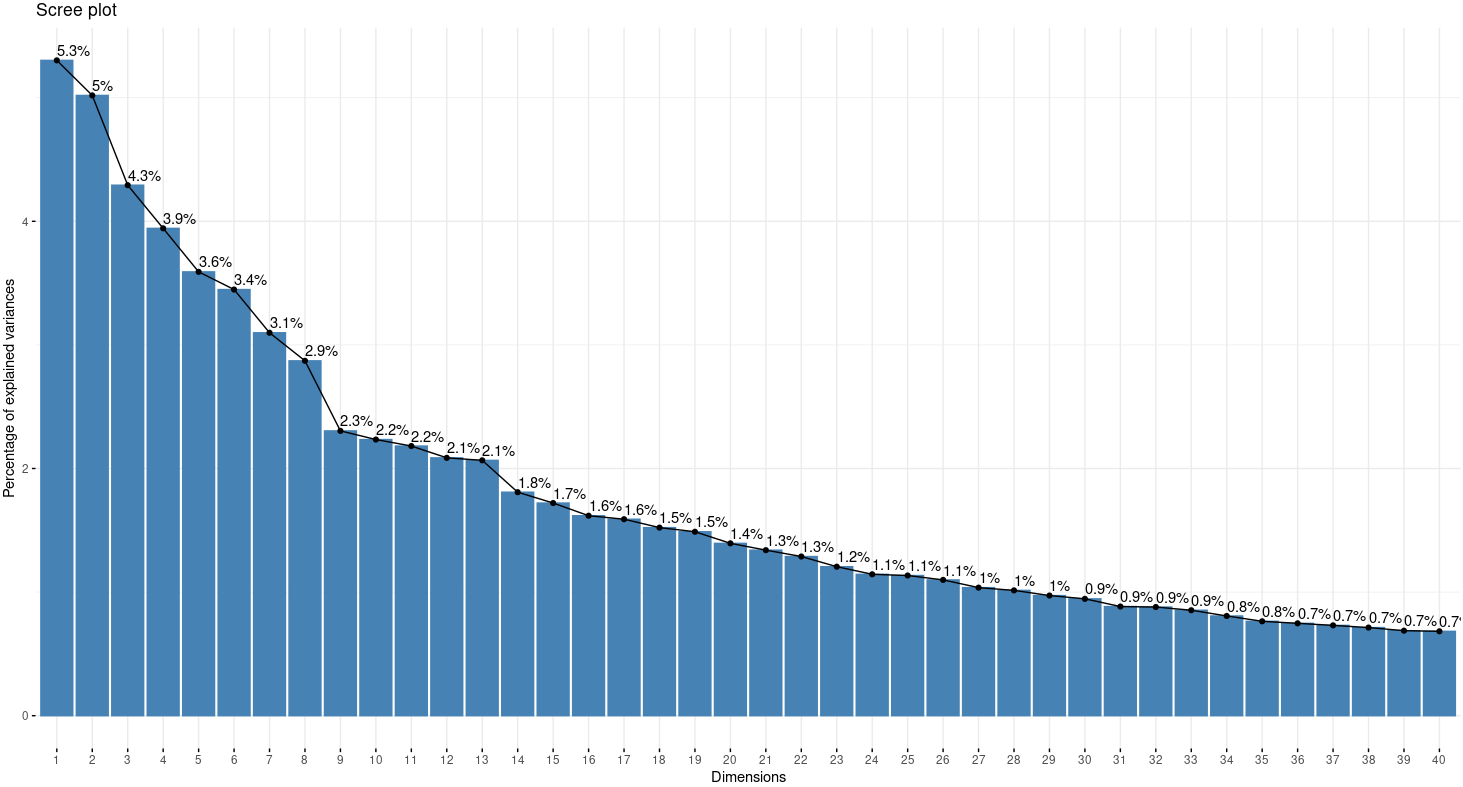
*

**Supplementary Figure 1:** Scree Plot, illustrating the variance of the principal components when PCA is applied to all self-supervised latent features (1 x 512).

*
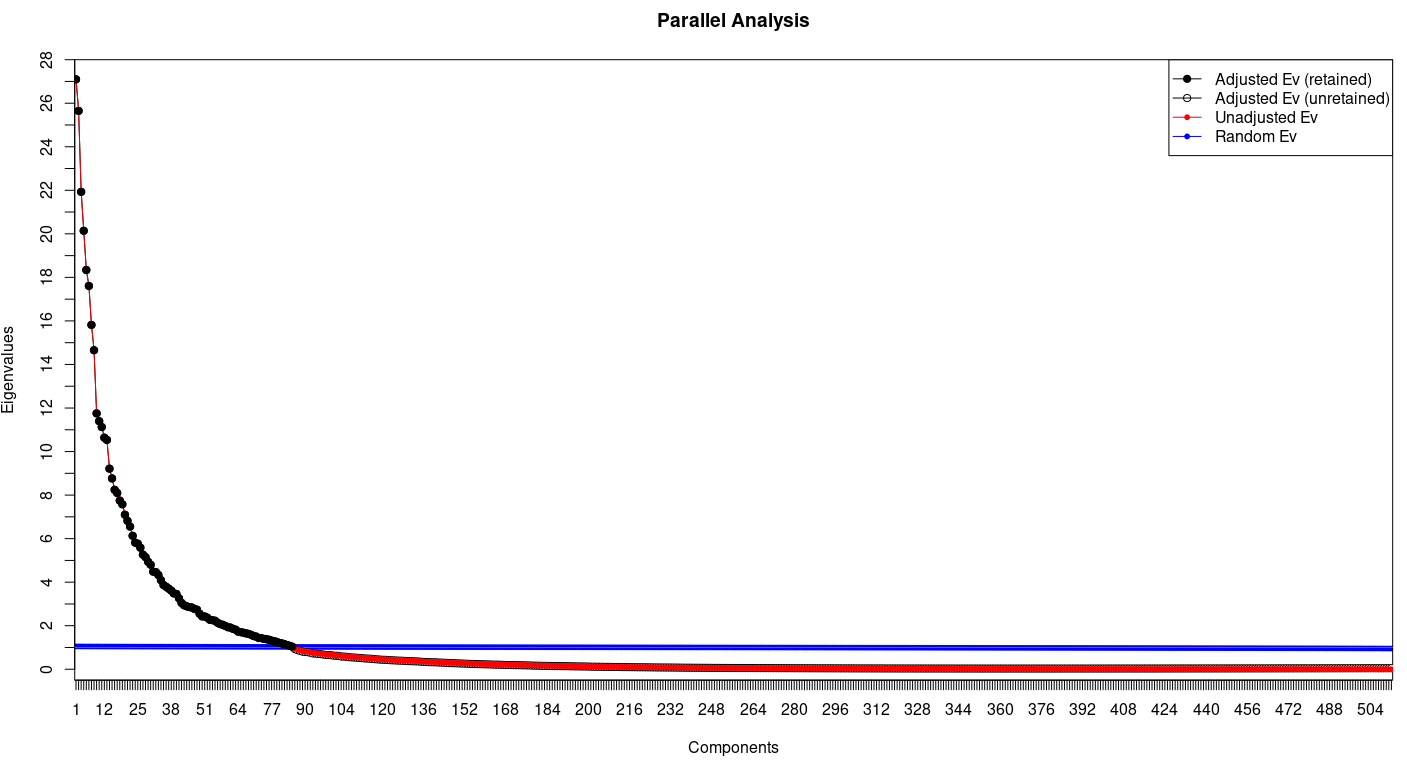
*

**Supplementary Figure 2:** Scree Plot to compare random generated eigenvalues (Ev) with the ones obtained by PCA to our dataset. PCs to retain are coloured in full black line, while the ones below the random generated line are discarded. This indicates 85 PCs should be retained.

# Clustering

After excluding the proportion of variance resulting from the same error (Horn’s parallel analysis), 85 PCs were retained in the cluster analysis to account for the data variance.

First, we assess the tendency to clustering for our dataset with Hopkins statistics. Values between 0.7-1 indicate clustered data. Our statistics revealed 1. This means that the clustering quality is high. Different clustering methods and numbers of clusters were assessed using python’s package Clustergram^1^.

**Clustering methods:**

1. k-means: The k-means clustering algorithm partitions the dataset into 'k' clusters by iteratively assigning data points to the cluster with the nearest mean. This method was employed to explore distinct groupings within our dataset.
2. Gaussian Finite Mixture Models: Gaussian finite mixture models were utilized as a probabilistic approach to clustering, assuming that the data is generated from a mixture of several Gaussian distributions. This method provides a flexible framework for capturing complex data patterns.

**Evaluation Metrics:**

To identify the optimal number of clusters and assess the quality of clustering, three metrics were employed:

1. Silhouette Plot: The Silhouette plot measures how similar an object is to its own cluster compared to other clusters. A higher silhouette score indicates better-defined clusters, assisting in determining the optimal number of clusters.
2. Calinski-Harabazs Plot: The Calinski-Harabazs index evaluates the ratio of between-cluster variance to within-cluster variance. A higher index suggests well-defined and separated clusters, aiding in the selection of the most suitable cluster number.
3. Davies-Bouldin Plot: The Davies-Bouldin index quantifies the compactness and separation between clusters. A lower index indicates better-defined and more separated clusters. This metric complements the evaluation of cluster quality.

Considering all the above comprehensive assessments, four robust clusters (Cluster 1, 2, 3 4) were identified within the dataset (Supplementary figure 3), with K-Means and a mini-batch size of 1000, enhancing the reliability of subsequent analyses and interpretations.

It is important to mention that Cluster 4 is only present in a relatively small subset of patients (n=24). Still, 22 out of these 24 patients exhibited significant coverage of Cluster 4 across more than 50% of their entire lung. Despite its limited patient representation, the decision to retain Cluster 4 was data-driven, having in mind the three evaluation metrics (Supplementary figure 3 A) and the *Clustergram* (Supplementary figure 3 B). These ensure the robustness and validity of including Cluster 4 in this analysis. From a clinical perspective, patients characterized by Cluster 4 demonstrated high percentages of gas trapping measures (31.3 ± 23.4%) and possessed an extensive smoking history (33 ± 12 years). This observation provides valuable insights into the distinct features associated with this specific group, despite its smaller representation in the overall dataset.


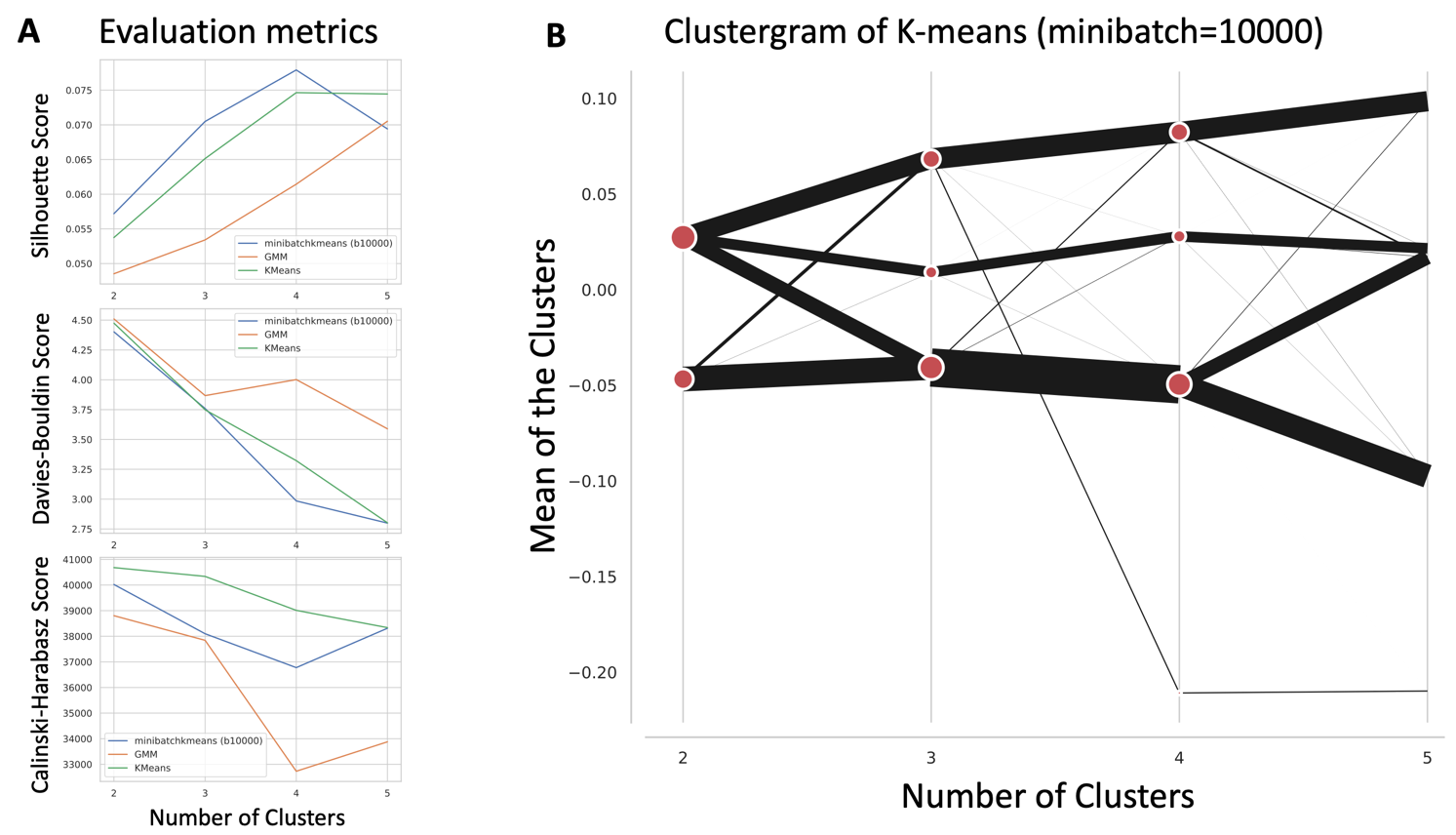


**Supplementary Figure 3.** Evaluation Metrics (A): Silhouette plot depicting its maximum to n=4 clusters with K-means minibatch=10000. Davies-Bouldin plot depicts its minimum to n=4 clusters with K-means minibatch=10000. Calinski-Harabazs plot depicts its minimum to n=4 clusters with Gaussian Mixture Models (GMM) and K-means minibatch=10000. (B) Clustergram graph of the k-means with minibatch=10000 depicting assigned cluster members as the number of clusters increases. The lines connecting points and their thickness represent observations moving between clusters. According to it, the optimal number of clusters is 4, as the steps afterwards do not show any difference in the branches.

# t-Distributed Stochastic Neighbor Embedding

To unravel the intricate patterns embedded in the self-supervised latent features of each region within the dataset and facilitate visual comprehension, we employed t-Distributed Stochastic Neighbor Embedding (t-SNE)^2^ as a non-linear dimensionality reduction technique. The openTSNE implementation^3^ was utilized for its efficiency and effectiveness. To enhance the preservation of the global structure, non-standard affinity methods were initially trained with the evaluation set of ^4,5^ and subsequently transformed to the current dataset (formerly the testset of ^4,5^). Optimal visual and computational results were achieved by initiating the training with a perplexity of 300 and an early exaggeration of 0.5. The transformation to the test set was performed with a perplexity of 50, ensuring a balance between preserving local and global relationships within the latent feature space. This approach not only allowed for a comprehensive understanding of the information encoded in the latent features but also optimized the visual representation, facilitating the interpretation of complex relationships across the dataset.

Supplementary figure 4 depicts the spatial representation from t-SNE colored by the gender, as to illustrate that the spatial distribution is not related to this factor, as a comparison to Figure 2 (main text).

*
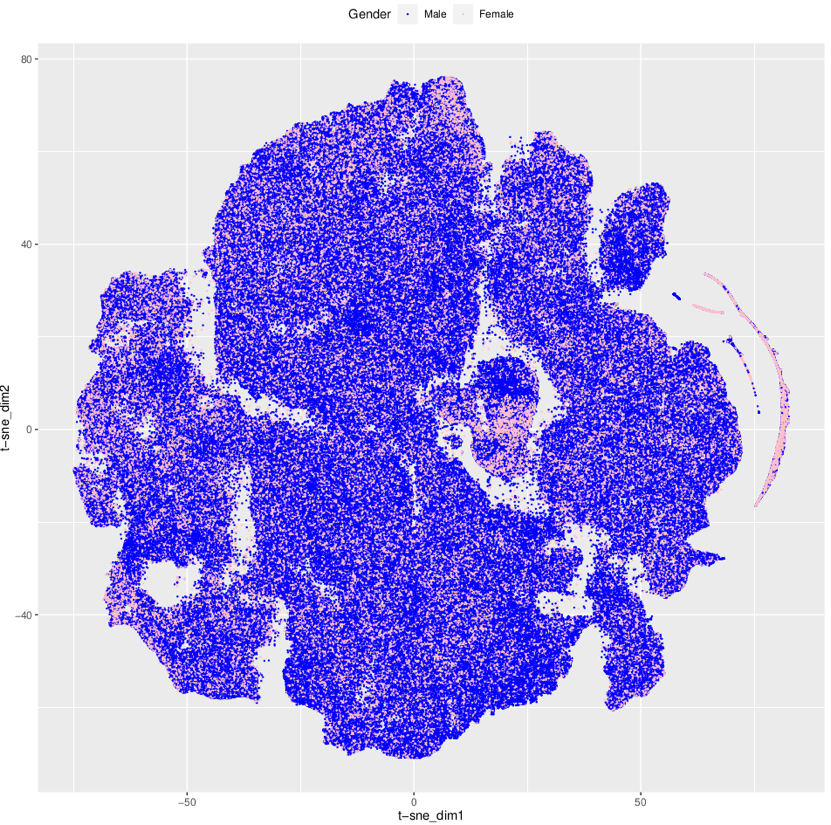
*

**Supplementary Figure 4:** t-SNE visualizations of the self-supervised contrastive latent space vectors. Each dot represents a region (3D ROI) or more specifically, an embedding of its latent representation into a two-dimensional space, and its color represents the gender (blue: male, pink: female)

# Relationships between PRM volumes, anomaly score, cluster groups in controls and GOLD 0 patients

As a means to study how the anomaly score and Cluster groups associate with clinical features in controls and GOLD 0 subjects, Pearson’s correlation coefficients were evaluated. This analysis was conducted on the same fashion as the main analysis conducted for all subjects, described in the Statistical Analysis Section of the main text.

Supplementary Table 1 depicts the Pearson correlation coefficient and correspondent corrected p-values (Holm-Bonferroni) for PRM volumes, anomaly score and cluster groups, referring to controls and GOLD 0. Overall, less significant correlations, with smaller effect sizes, were found in the healthy population. PRMfSAD showed a statistically significant correlations to FEV1/FVC, FRC, TLC, FRC/TLC and the smoking duration, from weak to large effect sizes. PRMEmph showed the same, with the exception for the smoking duration, but much smaller effect sizes. On the other hand, the anomaly score showed significant correlations to FEV1, FEV1/FVC, FRC, TLC, FRC/TLC, with weak to moderate effect sizes. Cluster correlations were fewer (mainly with FRC, TLC, FRC/TLC) with weak effect sizes. The anomaly score showed significantly higher correlations than PRM^fSAD^ for FEV1; and stronger than PRM^Emph^ for FEV1 and FRC/TLC. No significant differences were found between correlations of the anomaly score and PRM^fSAD^ for TLC; and between the anomaly score and PRM^Emph^ for FRC.

Supplementary Table 1: Correlation of PRM volumes, anomaly score and cluster groups with PFTs and Clinical Data for controls and GOLD patients. Confidence intervals are denoted in brackets, per each Pearson correlation coefficient. Correlations are colored according to Cohen’s effect size^6^ and only the significant ones are colored. None or very weak effect size is defined by |r|<0.3, weak 0.3<|r| <0.5, moderate 0.5<|r|<0.7 and large by |r|>0.7. PRM = Parametric Response Mapping; fSAD = functional Small Airway Disease; Emph = Emphysema; FEV1 = Forced Expiratory Volume in 1 second, FEV1/FVC = FEV/ Forced Vital Capacity, FRC = Functional Residual Capacity; TLC = Total Lung Capacity; BODE = Body mass index, air-flow Obstruction, Dyspnea, Exercise capacity; SGRQ = St George’s Respiratory Questionnaire; 6MWT = 6 minutes walking test. **** p>0.0001, *** p<0.001, ** p<0.01, *p<0.05.

| 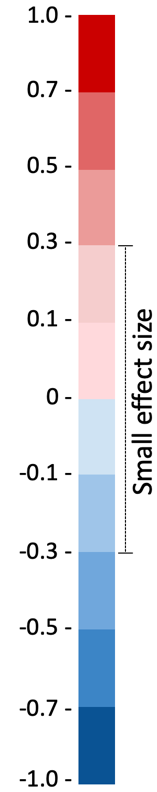 | **Method** | **Pulmonary Function Tests and Clinical Data for healthy patients only** | | | | | | | | |
| --- | --- | --- | --- | --- | --- | --- | --- | --- | --- | --- |
|  |  | **FEV1** | **FEV1/FVC** | **FRC** | **TLC** | **FRC/TLC** | **BODE** | **SGRQ** | **6MWT** | **Duration smoking** |
|  | **PRM** |  |  |  |  |  |  |  |  |  |
|  | **Healthy (%)** | 0.02 | 0.28**** | -0.66**** | -0.16*** | -0.72**** | 0.01 | 0.03 | 0.02 | -0.10* |
|  |  | (-0.07, 0.10) | (0.19, 0.37) | (-0.75, -0.58) | (-0.26, -0.06) | (-0.80, -0.64 ) | (-0.11, 0.12) | (-0.05, 0.11) | (-0.07, 0.12) | (-0.18, -0.01) |
|  | **Gas Trapping (%)** | -0.03 | -0.27**** | 0.67**** | 0.14** | 0.75**** | -0.01 | -0.03 | -0.03 | 0.10* |
|  |  | (-0.11, 0.06) | (-0.36, -0.18) | (0.59, 0.76) | ( 0.05, 0.24) | ( 0.69, 0.82) | (-0.12, 0.10) | (-0.11, 0.05) | (-0.13, 0.07) | (0.02, 0.18) |
|  | **Emphysema (%)** | 0.08 | -0.25**** | 0.34**** | 0.25**** | 0.16** | 0.01 | -0.01 | 0.03 | 0.02 |
|  |  | ( 0.00, 0.15) | (-0.32, -0.16) | (0.20, 0.47) | (0.15, 0.33) | (0.05, 0.27) | (-0.10, 0.13) | (-0.13, 0.11) | (-0.10, 0.16) | (-0.08, 0.12) |
|  | **cOOpD** |  |  |  |  |  |  |  |  |  |
|  | **Anomaly score** | -0.20**** | -0.11* | 0.27**** | -0.19*** | 0.50**** | 0.07 | 0.05 | -0.08 | 0.04 |
|  |  | (-0.29, -0.12) | (-0.20, -0.01) | ( 0.18, 0.36) | (-0.28, -0.09) | (0.42, 0.58) | (-0.03, 0.18) | (-0.06, 0.16) | (-0.18, 0.02) | (-0.06, 0.13) |
|  | **Clusters** |  |  |  |  |  |  |  |  |  |
|  | **1** | -0.06 | 0.06 | -0.32**** | -0.26**** | -0.26**** | 0.01 | -0.02 | 0.01 | -0.08 |
|  |  | (-0.16, 0.04) | (-0.05, 0.16) | (-0.44, -0.21) | (-0.35, -0.17) | (-0.40, -0.12) | (-0.08, 0.10) | (-0.11, 0.07) | (-0.09, 0.11) | (-0.17, 0.01) |
|  | **2** | 0.03 | 0.01 | -0.12* | 0.05 | -0.13* | 0.02 | -0.04 | 0.05 | -0.01 |
|  |  | (-0.07, 0.13) | (-0.09, 0.11) | (-0.23, -0.00) | (-0.05, 0.14) | (-0.25, 0.00) | (-0.08, 0.12) | (-0.14, 0.07) | (-0.06, 0.15) | (-0.10, 0.09) |
|  | **3** | -0.09 | -0.06 | 0.20**** | -0.04 | 0.25**** | 0.01 | 0.03 | -0.02 | -0.01 |
|  |  | (-0.17, 0.00) | (-0.16, 0.03) | (0.09, 0.32) | (-0.14, 0.06) | ( 0.13, 0.35) | (-0.10, 0.11) | (-0.08, 0.13) | (-0.12, 0.08) | (-0.10, 0.08) |
|  | **4** | 0.07 | 0.04 | 0.00 | 0.05 | -0.05 | -0.04 | 0.02 | -0.04 | 0.04 |
|  |  | (-0.03, 0.18) | (-0.07, 0.15) | (-0.06, 0.06) | (-0.03, 0.13) | (-0.11, 0.00) | (-0.09, 0.01) | (-0.08, 0.13) | (-0.15, 0.06) | (-0.05, 0.14) |

# References

1. Schonlau, M. The Clustergram: A Graph for Visualizing Hierarchical and Nonhierarchical Cluster Analyses. *The Stata Journal* **2**, 391–402 (2002).

2. van der Maaten, Laurens & Hinton, Geoffrey. Visualizing data using t-SNE. in.

3. Poličar, P. G., Stražar, M. & Zupan, B. *openTSNE: A Modular Python Library for t-SNE Dimensionality Reduction and Embedding*. http://biorxiv.org/lookup/doi/10.1101/731877 (2019) doi:10.1101/731877.

4. Almeida, S. D. *et al.* cOOpD: Reformulating COPD Classification on Chest CT Scans as Anomaly Detection Using Contrastive Representations. in *Medical Image Computing and Computer Assisted Intervention – MICCAI 2023* (eds. Greenspan, H. et al.) vol. 14224 33–43 (Springer Nature Switzerland, Cham, 2023).

5. Almeida, S. D. *et al.* Prediction of disease severity in COPD: a deep learning approach for anomaly-based quantitative assessment of chest CT. *Eur Radiol* (2023) doi:10.1007/s00330-023-10540-3.

6. *Statistical Power Analysis for the Behavioral Sciences*. (Elsevier, 1977). doi:10.1016/C2013-0-10517-X.
